# Newborn Screening for Spinal Muscular Atrophy in China Using DNA Mass Spectrometry

**DOI:** 10.3389/fgene.2019.01255

**Published:** 2019-12-17

**Authors:** Yiming Lin, Chien-Hsing Lin, Xiaoshan Yin, Lin Zhu, Jianbin Yang, Yuyan Shen, Chiju Yang, Xigui Chen, Haili Hu, Qingqing Ma, Xueqin Shi, Yaping Shen, Zhenzhen Hu, Chenggang Huang, Xinwen Huang

**Affiliations:** ^1^Department of Genetics and Metabolism, Children’s Hospital of Zhejiang University School of Medicine, National Clinical Research Center for Child Health, Hangzhou, China; ^2^Neonatal Disease Screening Center, Quanzhou Maternity and Children’s Hospital, Quanzhou, China; ^3^Department of Research and Development, Feng Chi Biotech Corp, Taipei, Taiwan; ^4^Department of Clinical Psychology, School of Health in Social Science, The University of Edinburg, Edinburg, United Kingdom; ^5^Department of Translational Medicine, Hangzhou Genuine Clinical Laboratory Co. Ltd, Hangzhou, China; ^6^Neonatal Disease Screening Center, Huaihua Maternal and Child Health Hospital, Huaihua, China; ^7^Neonatal Disease Screening Center, Jining Maternal and Child Health Family Service Center, Jining, China; ^8^Neonatal Disease Screening Center, Hefei Women and Children's Health Care Hospital, Hefei, China; ^9^Department of Pediatrics, Yancheng Maternity and Child Health Care Hospital, Yancheng, China; ^10^Research and Development Center, Zhejiang Biosan Biochemical Technologies Co., Ltd, Hangzhou, China

**Keywords:** spinal muscular atrophy, newborn screening, Agena iPLEX assay, MassARRAY genotyping, *SMN1*, *SMN2*

## Abstract

**Background:** Spinal muscular atrophy (SMA) is the most common neurodegenerative disorder and the leading genetic cause of infant mortality. Early detection of SMA through newborn screening (NBS) is essential to selecting pre-symptomatic treatment and ensuring optimal outcome, as well as, prompting the urgent need for effective screening methods. This study aimed to determine the feasibility of applying an Agena iPLEX SMA assay in NBS for SMA in China.

**Methods:** We developed an Agena iPLEX SMA assay based on the matrix-assisted laser desorption/ionization time-of-flight mass spectrometry, and evaluated the performance of this assay through assessment of 167 previously-genotyped samples. Then we conducted a pilot study to apply this assay for SMA NBS. The *SMN1* and *SMN2* copy number of screen-positive patients were determined by multiplex ligation-dependent probe amplification analysis.

**Results:** The sensitivity and specificity of the Agena iPLEX SMA assay were both 100%. Three patients with homozygous *SMN1* deletion were successfully identified and conformed by multiplex ligation-dependent probe amplification analysis. Two patients had two *SMN2* copies, which was correlated with severe SMA type I phenotype; both of them exhibited neurogenic lesion and with decreased muscle power. Another patient with four *SMN2* copies, whose genotype correlated with milder SMA type III or IV phenotype, had normal growth and development without clinical symptoms.

**Conclusions:** The Agena iPLEX SMA assay is an effective and reliable approach for population-based SMA NBS. The first large-scale pilot study using this assay in the Mainland of China showed that large-scale implementation of population-based NBS for SMA is feasible.

## Introduction

Spinal muscular atrophy (SMA) is an autosomal recessive neurodegenerative disorder and the leading genetic cause of infant mortality, with an incidence around 1 in 10,000 live births (Verhaart et al., 2017a; Verhaart et al., 2017b). About 95% of the SMA-affected patients are caused by the homozygous deletion of the survival of motor neuron 1 (*SMN1*) gene, resulting in deficiency of SMN protein (Lefebvre et al., 1995; Lefebvre et al., 1997). The *SMN1* gene is located in chromosome 5q13 containing *SMN2*. *SMN1,* and *SMN2* are highly homologous differing by only five nucleotides (Lefebvre et al., 1995; Cartegni and Krainer, 2002). The number of *SMN2* copies correlates with the severity of phenotypes. To be more specific, larger number of *SMN2* copies are associated with milder phenotypes (Mailman et al., 2002; Calucho et al., 2018).

Based on the age of onset and clinical severity, SMA is classified into five subtypes (0–IV) (Verhaart et al., 2017b). 60% of SMA-affected patients have severe SMA type I, and these patients usually progress to respiratory failure and even die within the first 2 years of life if they are left untreated (Kolb et al., 2017; Verhaart et al., 2017b). Early medical intervention on preventing motor neuron loss could generate maximal benefits to SMA-affected patients (Mendell et al., 2017). However, most patients are currently diagnosed with significant delay (Lin et al., 2015). Therefore, early detection of SMA through newborn screening (NBS) prior to the onset of neurodegeneration is essential to providing pre-symptomatic treatment and ensuring optimal outcome (Phan et al., 2015).

Given the serious clinical phenotype and high frequency of SMA, especially novel therapies drastically altering the course of disease and prolonging survival. Currently, SMA has been included into the recommended universal screening panel in the United States, and some states have lunched the SMA NBS program (Kanungo et al., 2018; Fabie et al., 2019). Furthermore, nationwide NBS for SMA are now being evaluated in other countries (Saffari et al., 2019). Thus, including SMA into NBS program has been highlighted recently, and NBS methods for SMA are needed more than ever.

Unlike conventional NBS practices, SMA does not have a specific biochemical analyte, thus DNA testing is undoubtedly the best approach. Several methods have been developed to detect the *SMN1* genotype in dried blood spot (DBS) samples, including real-time PCR (RT-PCR) (Pyatt and Prior, 2006; Taylor et al., 2015), competitive oligonucleotide priming PCR (Kato et al., 2015; Ar Rochmah et al., 2017), liquid microbead arrays (Pyatt et al., 2007), high-resolution melting analysis (Dobrowolski et al., 2012), and droplet digital PCR (ddPCR) (Vidal-Folch et al., 2018). However, standard methods for SMA NBS are still lacking. An ideal NBS assay must be cost-efficient, with high throughout, and easy to perform and automate. The Agena iPLEX assay is a MassARRAY genotyping platform based on the matrix-assisted laser desorption/ionization time-of-flight mass spectrometry (Calvo et al., 2010). It is one such platform that has successfully been utilized for identification of thousands of gene variations (Gabriel et al., 2009). The MassARRAY assay consists of an initial locus-specific PCR reaction, followed by single base extension using dideoxynucleotide terminators of a variant-specific oligonucleotide primer which anneals immediately upstream of the target site (Jurinke et al., 2004). Multiplexing application of the MassARRAY system, allowing for simultaneous assessment of multiple single-nucleotide polymorphisms (SNPs)/variants, is a cost-efficient way to augment high-throughput genotyping output. Herein, we described the development of an Agena iPLEX SMA assay to detect the homozygous *SMN1* deletion, which is applicable to SMA NBS. The performance of this assay was systemically studied and further evaluated by applying it to screen 29,364 newborns.

## Materials and Methods

### Subjects and Samples Preparation

To evaluate the sensitivity and specificity of the Agena iPLEX SMA assay design of this study, 167 newborns in September 2017, including one SMA case and 166 normal controls, were enrolled. The copy numbers of *SMN1* and *SMN2* genes of these 167 newborns were genotyped by using multiplex ligation-dependent probe amplification analysis (MLPA) method, which is the clinical golden-standard SMA approach. Pilot NBS was then performed to validate the application possibility of our design. The target screening sample size was calculated based on the incidence of 1:17,181 in Taiwan of China. Using PASS software (package 11.0), with permissible error of 0.03% and a two-sided 95% confidence interval, the necessary sample size needed to achieve statistical significance was 19,046 (**Supplementary File 1**). In order to increase the screening reliability, a total of 29,364 newborns from six hospitals were recruited for pilot NBS between March 2018 and June 2018. All newborns with expanded NBS results within the reference range. The participating hospitals include Children’s Hospital, Zhejiang University School of Medicine (n = 14,686), Quanzhou Maternity and Children’s Hospital (n = 2917), Huaihua Maternal and Child Health Care Hospital (n = 2905), Jining Maternal and Child Health Family Service Center (n = 2951), Yancheng Maternity and Child Health Care Hospital (n = 2965), and Anhui Women and Child Health Care Hospital (n = 2940). Blood samples were collected by heel stick and spotted on Whatman 903 filter paper. DBS samples of 167 and 29,364 newborns were sent to Hangzhou Genuine Clinical Laboratory (Hangzhou, Zhejiang, China) for SMA testing after the center had completed NBS. Genomic DNA was extracted from the DBS samples using a Qiagen Blood DNA mini kit (Qiagen, Hilden, Germany), and then preserved at −20°C refrigerator after measuring the concentration. DNA quality and quantity were confirmed using a NanoDrop 1000 UV-Vis spectrophotometer (Thermo Scientific, Wilmington, DE, USA). The concentration of DNA extracted from DBS was 12.513 ± 5.838 ng/µl. The project was approved by the Ethical Committee of Children’s Hospital, Zhejiang University School of Medicine. Written informed consents were obtained from parents of all the infants.

### Design of the Agena iPLEX SMA Assay

**Figure 1:**
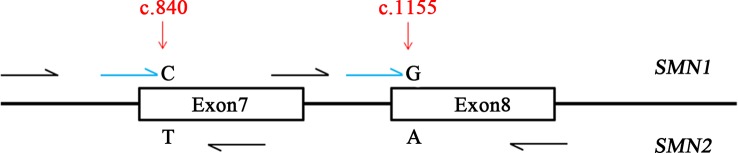
Design of the Agena iPLEX spinal muscular atrophy (SMA) assay. The iPLEX assay consists of a target-specific PCR reaction, followed by single-base extension using molecular weight-modified dideoxynucleotide terminators of an extension primer which anneals immediately upstream of the polymorphic site of interest two well-known positions of *SMN1* and *SMN2* genes (c.840 and c.1155) were used as targets for designing PCR and single-base extension primers. Black and blue colors indicate multiplex PCR and extension primers, respectively.

Due to the high similarity of *SMN1* and *SMN2* genes, two well-known positions (c.840 and c.1155) were used as targets for designing PCR and single-base extension (SBE) primers (**Figure 1**). The iPLEX assay consists of a target-specific PCR reaction, followed by SBE using molecular weight-modified dideoxynucleotide terminators of an extension primer which anneals immediately upstream of the polymorphic site of interest. Using matrix-assisted laser desorption/ionization time-of-flight mass spectrometry, the distinct mass of the extended primer identifies the SNP allele. The nucleotide of c.840 position is C and T in *SMN1* and *SMN2* genes, respectively. The nucleotide of c.1155 position is G and A in *SMN1* and *SMN2* genes, respectively. PCR and SBE primers (patent applications in progress) were designed for these two variants of *SMN1* and *SMN2* genes by using MassARRAY Assay Design 3.1 software (Agena, San Diego, CA) with 80 ≤ amplicon length (bp) ≤120 and 4,300 ≤ Mass Range (Da) ≤ 9,400. One PCR primer pair was designed for amplifying exon 7 of these two genes, and another was for exon 8. Beside these two variants of *SMN1* and *SMN2* genes, we also designed other polymorphic markers for sample identification (data not shown).

### MassARRAY-Based Genotyping

In the multiplex PCR reaction, DNA extracted from DBS was used to amplify an approximately 100 bp region targeting the two *SMN1/SMN2* variants of interest, including the c.840C/T and c.1155G/A. The 5 µl reaction containing 1 × PCR buffer (Agena), 2 mM MgCl_2_, 100 nM each amplification primer, 500 µM dNTPs, 25 ng DNA, and 1 U PCR enzyme (Agena). PCR conditions were 94°C for 4 min, followed by 45 cycles of 94°C for 20 s, 60°C for 30 s, 72°C for 60 s and a final incubation at 72°C for 3 min. Following the PCR, unincorporated dNTPs were inactivated by the addition of shrimp alkaline phosphatase (SAP) to the PCR reaction product [7 µl reaction containing 0.24× SAP buffer (Agena) and 0.07 U/µl SAP (Agena)]. Following SAP treatment, SBE onto the mutation site using the extension primers and assay-specific iPLEX terminator nucleotide mixes were performed (9 µl reaction containing 0.222× iPLEX buffer (Agena), 9 mM each iPLEX terminator nucleotide (Agena), 0.5–1 µM each extension primer (primer adjustment according to the primer mass based on regression method) and 1× iPLEX Pro DNA polymerase (Agena)]. PCR conditions were 94°C for 30 s, followed by 40 cycles of [94°C for 5 s, then 5 cycles of (58°C for 5 s, 80°C for 5 s)], and a final incubation at 72°C for 3 min. The mass spectrum from time-resolved spectra was retrieved by using a MassARRAY mass spectrometer (Agena), and each spectrum was then analyzed using SpectroTYPER software (Agena) to perform the genotype calling.

### MLPA Analysis

The *SMN1* and *SMN2* copy number were determined using MLPA [SALSA MLPA probemix P060 SMA (MRC-Holland, Amsterdam, Netherlands)]. The MLPA assay was performed according to the manufacture’s instruction. The PCR products were detected by ABL 3500XL capillary electrophoresis (Applied Biosystems, Foster City, CA, USA). The data were analyzed using the Coffalyzer software (version 3.5) to determine potential CNV (copy number variations) of exons. In brief, the dosage quotient values of 0, 0.4–0.65, 0.8–1.2, 1.3–1.65, and 1.75–2.15 indicates homozygous deletion, heterozygous deletion, normal copy number, heterozygous duplication, and homozygous duplication, respectively (**Supplementary files 2 and 3**).

## Results

### Validation Study

**Figure 2:**
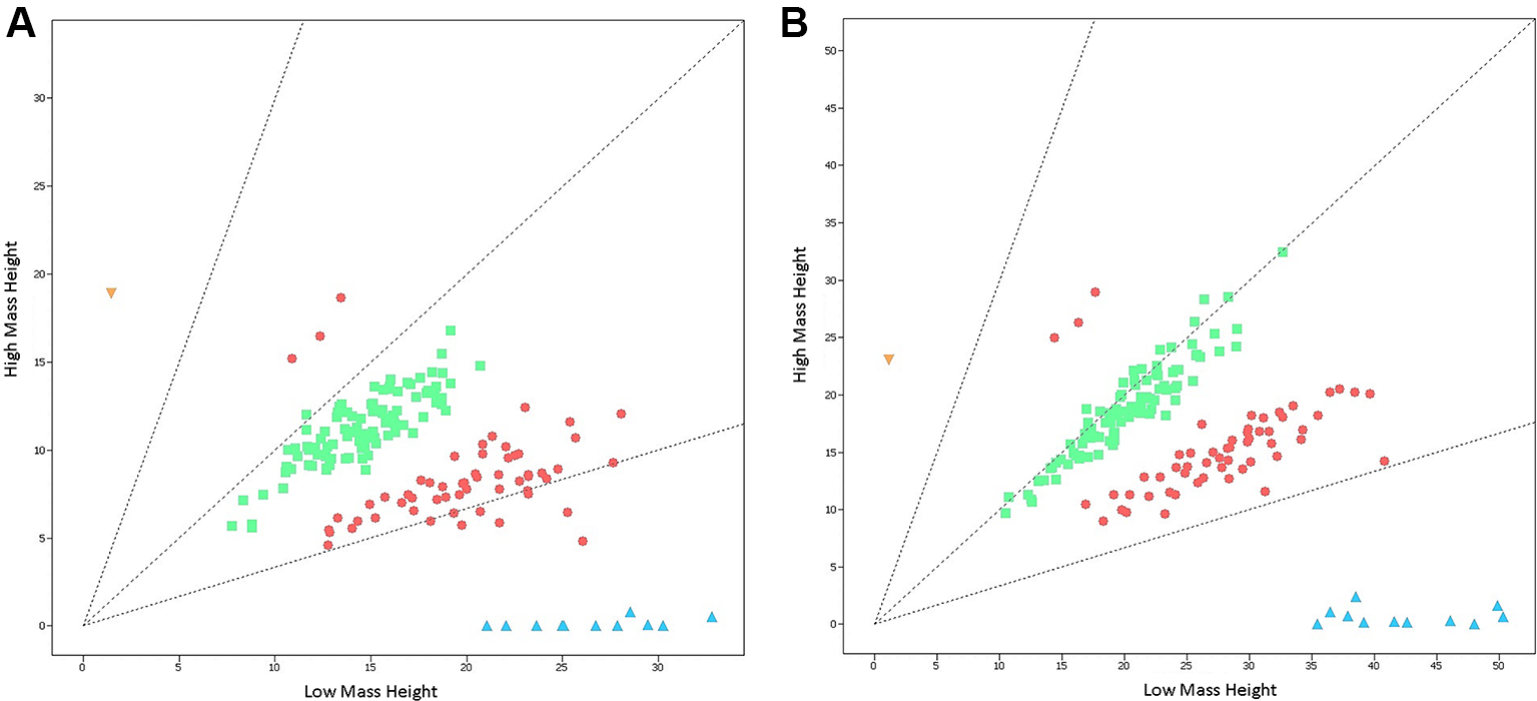
Cluster plots of 167 previously-genotyped samples detected by the Agena iPLEX SMA assay. **(A)** Cluster plots of samples at the nucleotide of c.840 in exon 7 of *SMN1* and *SMN2* genes. **(B)** Cluster plots of samples at the nucleotide of c.1155 in exon 8 of *SMN1* and *SMN2* genes. Low mass height represents the signal strength of *SMN1*, while high mass height represents the signal strength of *SMN2*. The yellow triangle (*SMN1*:*SMN2* = 0:2) indicate SMA-affected positive sample with homozygous *SMN1* deletion. The red circles (*SMN1*:*SMN2* = 2:1 or 1:2), the green squares (*SMN1*:*SMN2* = 2:2), and the blue triangles (*SMN1*:*SMN2* = 2:0) indicate normal non-homozygous deletion samples.

The Agena iPLEX SMA assay was validated in a double-blind testing of 167 previously-genotyped DBS samples with known *SMN1* and *SMN2* copy numbers. There was a clear distinction between positive samples with homozygous *SMN1* deletion and normal individuals (**Figure 2**). The only case of SMA-affected patient was accurately identified from 167 samples of different genotypes, and the analytical results showed 100% concordance. Thus, the sensitivity and specificity of this screening assay were both 100%.

### NBS for SMA

**Figure 3:**
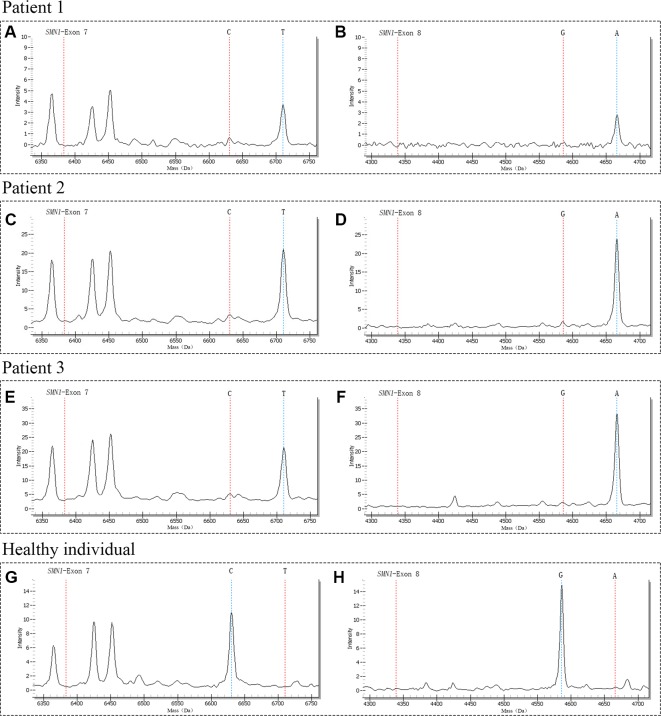
The mass spectra of SMA-affected positive patients and healthy individual. Patient 1 had only one peak at 6710.3 Da **(A)** and 4665.9 Da **(B)** respectively, indicating the homozygous deletions of *SMN1* exons 7 and 8; Patient 2 had only one peak at 6710.3 Da **(C)** and 4665.9 Da **(D)** respectively, indicating the homozygous deletions of *SMN1* exons 7 and 8; Patient 3 had only one peak at 6710.3 Da **(E)** and 4665.9 Da **(F)** respectively, indicating the homozygous deletions of *SMN1* exons 7 and 8; The control of healthy individual had only one peak at 6630.3 Da **(G)** and 4586.0 Da **(H)** respectively, indicating with normal copy number of *SMN1*. The red vertical dotted lines on the left indicate the mass of unextension primer, the red and blue vertical dotted lines on the right indicate the mass of primer after extension.

**Figure 4:**
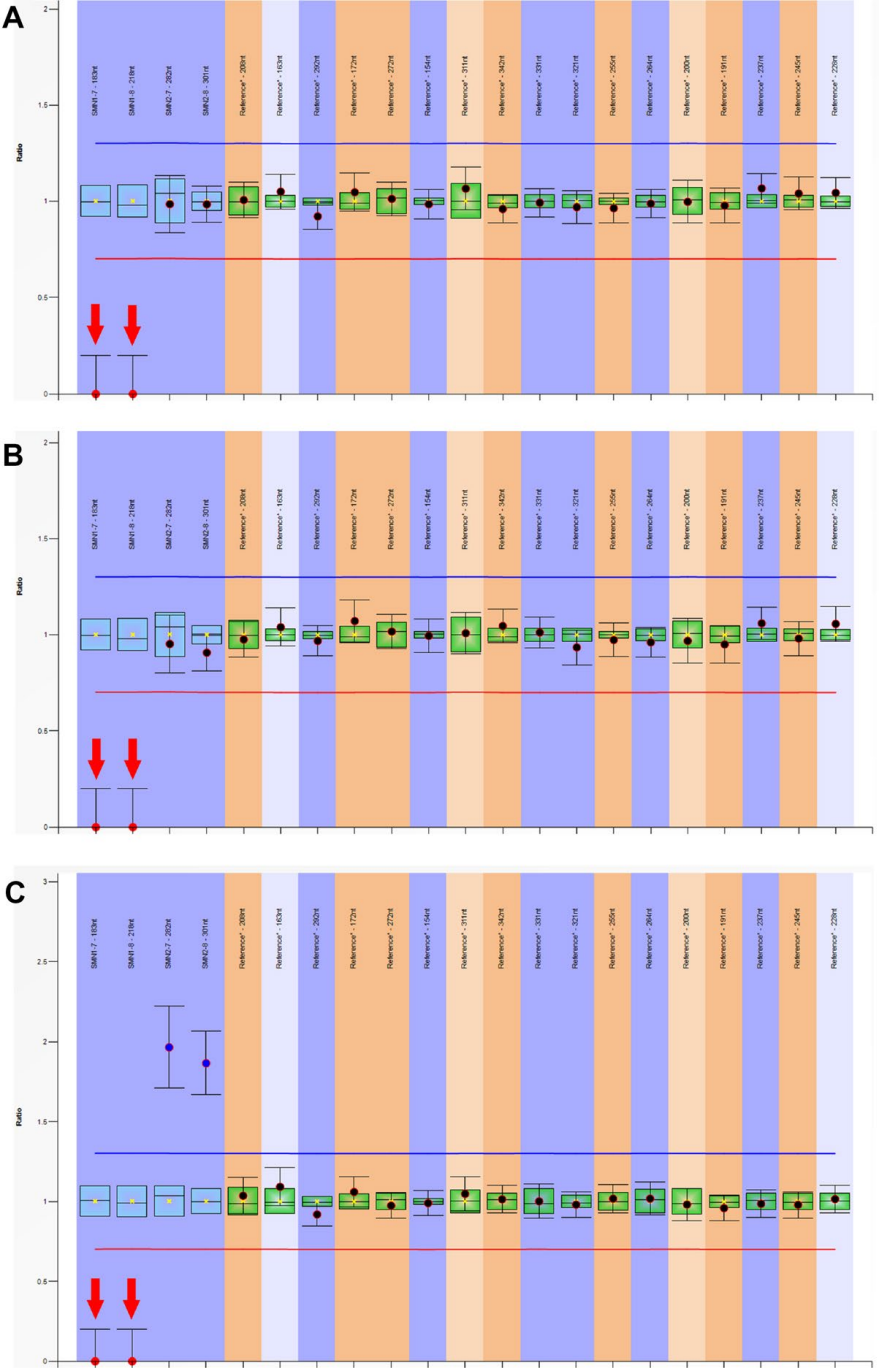
Multiplex ligation-dependent probe amplification (MLPA) analysis of *SMN1* and *SMN2* genes. The dosage quotient (DQ) values of 0, 0.4–0.65, 0.8–1.2, 1.3–1.65, and 1.75–2.15 indicates homozygous deletion, heterozygous deletion, normal copy number, heterozygous duplication, and homozygous duplication, respectively. The homozygous deletions of *SMN1* exons 7 and 8 were detected in three patients, **(A)** patient 1 (*SMN1*:*SMN2* = 0:2); **(B)** patient 2 (*SMN1*:*SMN2* = 0:2); **(C)** patient 3 (*SMN1*:*SMN2* = 0:4).

A total of 29,364 individuals were screened, and three newborns with SMA were identified. All these positive patients were confirmed to have homozygous deletions of *SMN1* exons 7 and 8 by MLPA, yielding an incidence of 1:9788 (**Figures 3** and **Figure 4**). Among which two patients (patients 1 and 2) had two *SMN2* copies, which was correlated with severe SMA type I phenotype. Both patients exhibited neurogenic lesion and showed decreased muscle power. While another patient (patient 3) had four *SMN2* copies, with genotype correlated with milder SMA type III or IV phenotype; the patient had normal growth and development without symptoms during the latest follow-up visit (**Table 1**).

**Table 1:** Newborn screening results for spinal muscular atrophy.

Patients no.	Gender	Province/city	Age of onset	MassARRAY-based genotype	*SMN1*:*SMN2* copies by MLPA	Clinical features (Classification)	Evolution
1	Female	Zhejiang	<3 months	Homozygous deletions of *SMN1* exons 7 and 8	0:2	Exhibited neurogenic lesion and with decreased muscle power (I)	Died at 5 months old
2	Male	Zhejiang	<3 months	Homozygous deletions of *SMN1* exons 7 and 8	0:2	Exhibited neurogenic lesion and with decreased muscle power (I)	Severe
3	Male	Hefei	Not found	Homozygous deletions of *SMN1* exons 7 and 8	0:4	Normal (III or IV)	Well with normal growth and development

MLPA, multiplex ligation-dependent probe amplification.

## Discussion

The Agena iPLEX assay is a robust platform for the detection of many genetic variations, which has been approved for clinical diagnosis. In this study, through taking full advantage of the homologous sequences of *SMN1* and *SMN2*, we translated deletions to mutations and successfully established an Agena iPLEX SMA assay to detect homozygous *SMN1* deletion. The sensitivity and specificity of the Agena iPLEX SMA assay were both 100%. Then, we conducted a pilot study to apply this assay in SMA NBS. Three patients having SMA with homozygous deletions of *SMN1* exons 7 and 8 were successfully identified and diagnosed by MLPA. The sensitivity of this assay applied in NBS was 95%, because this assay detected only 95% of homozygous *SMN1* deletion mutations.

Although there are several technologies available for SMA NBS, whereas each method has its limitations. For example, ddPCR is relatively costly and the instrument is scarce; liquid microbead array is rarely used in less developed regions; RT-PCR requires normalization or standard curves; competitive oligonucleotide priming PCR involves multiple steps of post-PCR manipulations leading to labor intensive; and high-resolution melting analysis requires an experienced researcher to optimize the reaction conditions and analyze the data. Therefore, we explored the use of Agena iPLEX SMA assay for the identification of homozygous *SMN1* deletion. This assay is automated with easier operation, and it is capable to analyzing larger batches of samples daily. Meanwhile, the results are easy to interpreted and even implemented in small/medium-sized NBS laboratories. Moreover, many quality reference materials such as gender and SNPs can be built in for sample traceability. Furthermore, the Agena iPLEX SMA assay has great adaptability with DBS specimens, and previous studies have demonstrated the successful use of Agena iPLEX assay for Fabry NBS (Lu et al., 2018). Last but not least, the Agena iPLEX SMA assay has the advantage of flexibility in that more diseases can be incorporated into the current assay, such as integrated severe combined immunodeficiency, the first DNA-based NBS condition that was detected by measuring the T-cell receptor excision circles, for prospective molecular screening. Thus, the Agena iPLEX SMA assay has great potential to be widely applied in NBS for SMA.

NBS is tasked with differentiating affected patients from healthy individuals, however, some carriers may be identified in actual work. The identification of carriers is problematic because it increases unnecessary stress to the parents. The approach in this study was designed to exclusively detect only homozygous *SMN1* deletion and the unwanted detection of SMA carriers can be effectively avoided. Of note is that a small proportion (5% of total) of *SMN1* intragenic variants cannot be detected through the current Agena iPLEX SMA assay, which needs to be clarified during the process of informed consent.

There were several pilot studies on NBS of SMA and the feasibility of DNA-based NBS for SMA has been demonstrated. For instance, Jennifer et al. used a multiplex TaqMan RT-PCR to screen 3826 newborns in New York. In this research, one neonate with SMA was successfully detected and the study was accepted by these families (Kraszewski et al., 2018). Chien et al. (2017) used a RT-PCR combined with ddPCR as second-tier testing screened 120,267 newborns in Taiwan of China, 15 newborns were tested positive for primary screening, and 7 patients were finally diagnosed as SMA. Another study conducted by Thomas et al. using liquid microbead arrays screened 40,103 DBS samples, four positive patients with SMA were successfully identified (Prior et al., 2010). The current study revealed that Agena iPLEX SMA assay is applicable for SMA NBS; and the frequency of SMA in our population is 1:9788, which is similar to 1:10,026 in Ohio and higher than 1:17,181 in Taiwan of China (Prior et al., 2010; Chien et al., 2017). Therefore, Agena iPLEX SMA assay has high specificity and sensitivity, and at high throughput, is an effective and reliable approach for population-based SMA NBS.

This is the first large-scale study of NBS for SMA in the Mainland of China, two patients with severe SMA type I were detected. However, because the first U.S. Food and Drug Administration approved drug for SMA therapy, Spinraza, which was not available in China, both patients in this series were only eligible for adjuvant therapy such as nutritional and respiratory support (Finkel et al., 2017; Ottesen, 2017; Gidaro and Servais, 2019). Consequently, the treatment of these two patients were not effective, one patient died and the other is still under poor prognosis. But more importantly, the detection of SMA is of great significance for the genetic counseling and reproductive guidance of these families, especially due to the limitation that carrier screening has not been widely carried out in China. China has long-term faced many challenges in the screening and treatment of SMA, including low level of public awareness of the disease, lack of genetic testing channels, and effective treatment drugs, as well as lack of standardized long-term follow-up and management (Ke et al., 2019). These challenges have seriously hindered the development of diagnosis and treatment of SMA in China, and have brought many difficulties to SMA-affected patients and their families. Fortunately, Spinraza was introduced in China in April 2019, indicating that the incurable dilemma of SMA should be resolved. Simultaneously with the launch of Spinraza, The Diagnosis and Treatment Center for SMA was founded in China and will provide better medical services for Chinese SMA-affected patients. In addition, the Chinese government is currently paying increasing attention to SMA and has formulated a series of policies which benefit patients and their families. The current study demonstrated the feasibility of SMA NBS, which will undoubtedly advance the progress of SMA in China.

In summary, we developed a simple and automated high-throughput SMA assay for SMA NBS. The assay is rapid (7 h from DNA preparation to data report), inexpensive (the running cost is approximately $3/sample), and with high specificity and sensitivity. Thus, the assay could be recommended as an efficient tool for SMA NBS. The first large-scale pilot study using this assay in the Mainland of China showed that large-scale implementation of population-based NBS for SMA is feasible. We believe this work could advance NBS for SMA.

## Data Availability Statement

All datasets generated for this study are included in the article/**Supplementary Material**.

## Ethics Statement

The project was approved by the Ethical Committee of Children's Hospital, Zhejiang University School of Medicine (reference number: 2018-IRB-077).

## Author Contributions

YL performed experimental work, paper writing and drafting; C-HL designed the study and participated in paper editing; XY carried out the mutation analysis and paper editing; LZ carried out the genetic tests, mutation analysis and paper editing; CH participated in manuscript preparation; JY, YuS, CY, XC, HH, QM, XS, YaS and ZH followed the patients and collected the clinical data; XH was mentors who designed and guided the research study. All authors read and approved the final manuscript.

## Funding

This work was supported by the National Key Research and Development Program of China (grant number 2018YFC1002200).

## Conflict of Interest

CL is currently an employee of Feng Chi Biotech Corp. LZ is currently an employee of Genuine Diagnostics Company. CH is currently an employee of Zhejiang Biosan Biochemical Technologies Corp.

The remaining authors declare that the research was conducted in the absence of any commercial or financial relationships that could be construed as a potential conflict of interest.
