# Supplementary material for: Newborn Screening for Spinal Muscular Atrophy in China Using DNA Mass Spectrometry
Source: Front Genet. 2019 Dec 17;10:1255. doi: 10.3389/fgene.2019.01255 (PMC6928056; doi:10.3389/fgene.2019.01255)

Confidence Intervals for One Proportion - New

Numeric Results for Two-Sided Confidence Intervals for One Proportion
Confidence Interval Formula: Exact (Clopper-Pearson)

Confidence Level	Sample Size (N)	Target Width	Actual Width	Proportion (P)	Lower Limit	Upper Limit	Width if P = 0.5
0.950	19046	0.000	0.000	0.000	0.000	0.000	0.014

References

Fleiss, J. L., Levin, B., Paik, M.C. 2003. Statistical Methods for Rates and Proportions. Third Edition. John Wiley & Sons. New York.
Newcombe, R. G. 1998. 'Two-Sided Confidence Intervals for the Single Proportion: Comparison of Seven Methods.' Statistics in Medicine, 17, pp. 857-872.

Report Definitions

Confidence level is the proportion of confidence intervals (constructed with this same confidence level, sample size, etc.) that would contain the population proportion.
N is the size of the sample drawn from the population.
Width is the distance from the lower limit to the upper limit.
Target Width is the value of the width that is entered into the procedure.
Actual Width is the value of the width that is obtained from the procedure.
Proportion (P) is the assumed sample proportion.
Lower Limit is the lower limit of the confidence interval.
Upper Limit is the upper limit of the confidence interval.
Width if P = 0.5 is the maximum width for a confidence interval with sample size N.

Summary Statements

A sample size of 19046 produces a two-sided 95% confidence interval with a width equal to 0.000 when the sample proportion is 0.000.

Confidence Intervals for One Proportion - New

Chart Section

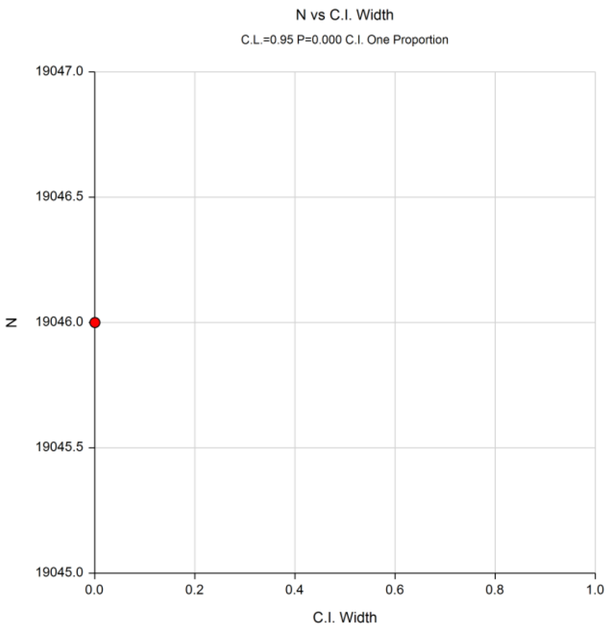

Supplement: Supplementary file 1 [file DataSheet_1.pdf]
