# Supplementary material for: Newborn Screening for Spinal Muscular Atrophy in China Using DNA Mass Spectrometry
Source: Front Genet. 2019 Dec 17;10:1255. doi: 10.3389/fgene.2019.01255 (PMC6928056; doi:10.3389/fgene.2019.01255)
Supplement: Supplementary file 2 [file DataSheet_2.pdf]

Product Description SALSA® MLPA® probemix P060-B2 SMA

To be used with the MLPA General Protocol.

Version B2. As compared to version B1, the 88 and 96 nt DNA denaturation control fragments have been replaced (QDX2). For a complete product history see page 9.

Catalogue numbers:
- **P060-025R:** SALSA® MLPA® probemix P060 SMA, 25 reactions.
- **P060-050R:** SALSA® MLPA® probemix P060 SMA, 50 reactions.
- **P060-100R:** SALSA® MLPA® probemix P060 SMA, 100 reactions.

To be used in combination with a SALSA® MLPA® reagent kit, available for various number of reactions. MLPA reagent kits are either provided with FAM or Cy5.0 dye-labelled PCR primer, suitable for Applied Biosystems and Beckman capillary sequencers, respectively (see www.mlpa.com).

Certificate of Analysis: Information regarding storage conditions, quality tests, and a sample electropherogram from the current sales lot is available at www.mlpa.com.

Precautions and warnings: For professional use only. Always consult the most recent product description AND the MLPA General Protocol before use: www.mlpa.com. It is the responsibility of the user to be aware of the latest scientific knowledge of the application before drawing any conclusions from findings generated with this product.

Intended use: This SALSA® MLPA® probemix P060 SMA is an in vitro diagnostic (IVD)[1] or research use only (RUO) assay for the detection of copy number changes of exons 7 and 8 of SMN1 and SMN2 for patient diagnosis and carrier testing of spinal muscular atrophy (SMA).

This assay can be used with human DNA derived from peripheral blood, (un)cultured amniotic fluid obtained in week 16 of the pregnancy or later and free from blood contamination, (un)cultured chorionic villi free from maternal contamination, or fetal blood. The results of this test should be interpreted by a clinical molecular geneticist or equivalent.

[1]Please note that this probemix is for In Vitro Diagnostic use (IVD) in the countries specified at the end of this product description. In all other countries, the product is for Research Use Only (RUO).

Clinical background: Spinal muscular atrophy (SMA) is a neuromuscular disorder characterised by degeneration of the anterior horn cells of the spinal cord, leading to symmetrical muscle weakness and atrophy. The estimated incidence of SMA is 1:6,000-1:10,000: The second most common lethal autosomal recessive disorder in Caucasians, after cystic fibrosis (Ben-Shachar et al. 2011, Smith et al. 2007). SMA is usually divided into four clinical groups based on age of onset and maximum function obtained (type I, OMIM# 253300; type II, OMIM# 253550; type III, OMIM# 253400; and type IV, OMIM# 271150).

Two (highly-similar) genes play a pivotal role in SMA: SMN1 and SMN2. Most individuals have two copies of each gene. The SMA region on 5q13.2, containing the telomeric SMN1 and the centromeric SMN2, is a complicated inverted repeat area displaying high instability, leading to frequent deletions and gene conversions. SMN1 and SMN2 can only be distinguished by two single nucleotide differences: one in exon 7 and one in exon 8. The single nucleotide difference in exon 7 of SMN2 affects mRNA splicing resulting in an altered SMN protein with a limited half-life and function.

95-98% of SMA patients (51% of South African black SMA patients) show homozygous deletion of at least exon 7 of the telomeric SMN1 gene (Labrum et al. 2007). The remaining 3-5% present compound heterozygosity with a point mutation on one chromosome and a deletion/gene conversion on the other. Such a point mutation will not be detected by this P060 SMA MLPA assay and should be identified by sequencing. In a very small number of patients the SMN1 defect is a copy number change of SMN1 exons 1-

6 which may be detectable with the P021 SMA MLPA probemix (Arkblad et al. 2006). The great majority of SMA carriers can be identified by the presence of only a single SMN1 exon 7 copy. The one copy frequency in the US is estimated to be 1:37 for Caucasians, 1:46 for Ashkenazi Jews, 1:56 for Asians, 1:91 for African-Americans and 1:125 for Hispanics. Additionally, about 3-8% of SMA carriers (27% of African Americans) have two SMN1 copies on one chromosome and 0 copies on the other (2+0) (Alias et al. 2014, Ben-Shachar et al. 2011, Hendrickson et al. 2009, Miskovic et al. 2011, Smith et al. 2007). Dosage analysis cannot determine the difference between '1+1' and '2+0' (silent carriers) arrangements. Both situations are simply detected as having two SMN1 copies leading to false negative results. A thorough molecular analysis should be performed in parents and blood relatives of SMA patients when initial results indicate two SMN1 copies. Recently, Luo et al. (2014) reported that a haplotype block specific for SMN1 duplications is present in a large percentage of Ashkenazi Jews and in other ethnic groups. Identifying this haplotype will help distinguish silent carriers. If interested in more information regarding the identification of this haplotype, please contact info@mlpa.com.

The SMN2 copy number is very variable with only 60-70% of individuals having two copies. Provided that at least one functional SMN1 copy is present, complete absence of the centromeric SMN2 gene seems to have no clinical consequences. However, determining the SMN2 copy number is important for SMA patients: the more SMN2 copies, the less severe the disease is expected to be. More information on spinal muscular atrophy can be found in http://www.ncbi.nlm.nih.gov/books/NBK1352/.

Gene structure: SMN1 and SMN2, each having 9 exons, are part of a 500 kb inverted duplication on chromosome 5q13. SMN1 LRG_676 is available at http://www.lrg-sequence.org/ and is identical to Genbank NG_008691.1. SMN2 LRG_677 is pending approval and is identical to GenBank NG_008728.1.

Transcript variants: SMN1 – three transcript variants have been described: www.ncbi.nlm.nih.gov/gene/6606. SMN1 transcript variant d (NM_000344.3, 1641 bp, coding sequence 164-1048) is the longest, most predominant transcript. SMN1 transcript variant a (NM_001297715.1) and transcript variant b (NM_022874.2) are shorter variants than compared to variant d. SMN2 – four transcript variants have been described: www.ncbi.nlm.nih.gov/gene/6607. SMN2 transcript variant d (NM_017411.3, 1634 bp, coding sequence 164-1048) encodes the longest transcript. SMN2 transcript variant a (NM_022875.2) lacks an alternate exon in the 3' CDS compared to variant d and is thought to be the predominant transcript. SMN2 transcript variant b (NM_022876.2) and transcript variant c (NM_022877.2) are shorter than transcript variant d.

Exon numbering: The exon numbering used in this P060-B2 SMA product description and in the P060-B2 SMA lot-specific Coffalyser.Net analysis sheet is the traditional exon numbering (exons 1, 2a, 2b, and 3-8). **Please note that the SMN1 and SMN2 exon numbering is different in the SMN1 and SMN2 LRG sequence and in the NCBI NG_008691.1 and NG_008728.1 reference sequence.**

P060-B2 probemix content: This SALSA® MLPA® probemix P060 SMA contains 21 MLPA probes with amplification products between 154 and 342 nt (Table 2) including 2 probes each for SMN1 and SMN2 (Table 2) and 17 reference probes that detect sequences outside this region. The identity of the genes detected by the reference probes is available online (www.mlpa.com).

- The **SMN1 Exon 7 probe 14919-L17081** (183 nt) is the most important probe as it can be used to determine SMN1 copy number, which is important for deducing SMA carrier status. This probe is specific for SMN1 and will give no significant signal on SMN2. The probe has its ligation site at the C-to-T transition in exon 7, which is the site that affects RNA splicing in SMN2.

- The **SMN1 Exon 8 probe 14881-L17082** (218 nt) is able to distinguish between SMN1 and SMN2 at exon 8 (G-to-A transition). The signal of this probe indicates the copy number of SMN1 exon 8. In approximately 95% of the samples, the copy number detected by the SMN1 exon 7 probe and the SMN1 exon 8 probe is identical. This SMN1 exon 8 probe cannot be used to quantify the number of SMN1 copies, as an exon 8 mutation will still result in a functional protein. Only the SMN1 exon 7 probe should be used to determine the SMN1 copy number. In the majority of the remaining 5% of samples, gene conversion between SMN1 and SMN2 has resulted in a chimeric gene containing the SMN1 exon 7 sequence and the SMN2 exon 8 sequence. Such a hybrid gene results in a functionally identical protein to the SMN1 protein.

- The **SMN2 Exon 7 probe 14921-L17083** (282 nt) identifies the SMN2 copy number, which is important for SMA patients, but has no influence on SMA carrier status.

- The **SMN2 Exon 8 probe 14878-L17084** (301 nt) confirms the results obtained with the SMN2 exon 7 probe in most individuals. In case the copy number detected by this exon 8 probe does not correspond to that found by the exon 7 probe, only the exon 7 probe should be used to determine SMN2 copy number.

The summary of these findings and what they mean for carrier/patient status can be found in Table 1.

This probemix contains nine quality control fragments generating amplification products between 64 and 105 nt: four DNA Quantity Fragments (Q-fragments), three DNA Denaturation Fragments (D-fragments), and one chromosome X and one chromosome Y-specific fragment (Table 2). The Q-fragments are only visible when less than 100 ng sample DNA is used. Low signal of the 88 or 96 nt fragment indicates incomplete DNA denaturation. More information on how to interpret observations on these control fragments can be found in the MLPA General Protocol.

MLPA technique: The principles of the MLPA technique (Schouten et al. 2002) are described in the MLPA General Protocol (www.mlpa.com).

MLPA technique validation: Internal validation of the MLPA technique using 16 DNA samples from healthy individuals is required, in particular when using MLPA for the first time, or when changing the sample handling procedure, DNA extraction method or instruments used. This validation experiment should result in a standard deviation <0.10 for all reference probes over the experiment.

Required specimens: Purified DNA from peripheral blood, (un)cultured amniotic fluid obtained in week 16 of the pregnancy or later and free from blood contamination, (un)cultured chorionic villi free from maternal contamination, or fetal blood. Samples should be free from impurities know to affect MLPA reactions. For more information please refer to the section on DNA sample treatment found in the MLPA General Protocol.

Reference samples: The choice of reference samples is important for the correct determination of the SMN1 and SMN2 copy numbers. MRC-Holland is not able to provide reference DNA samples. One reason is that for MLPA reactions, the reference DNA samples should be derived from the same tissue type, handled using the same procedure, and prepared using the same DNA extraction method as the patient samples. It is strongly advised to first make a selection of suitable reference samples with known copy numbers before SMA testing is started. In particular, suitable reference samples are essential when testing patients with African ancestry. One method of doing this is to test a number (e.g. 16) of healthy individuals who are from families without a history of SMA for two copies of both SMN1 and SMN2. More information regarding the selection and use of reference samples can be found in the MLPA General Protocol.

If there is any doubt regarding the SMN1/SMN2 copy number of your reference samples, SD019 (SALSA Reference DNA) can facilitate the identification of suitable reference DNA samples. Please note that SD019 is for research use only (RUO). We recommend the use of SD019 only for initial experiments on DNA samples from healthy individuals with the intention to identify suitable reference DNA samples. We do not recommend it for use in all experiments. Please see the SD019 product description on www.mlpa.com for further details.

Positive control DNA samples: MRC-Holland cannot provide positive DNA samples. Inclusion of a positive sample in each experiment is recommended.

Performance characteristics: The expected number of Caucasian SMA patients that can be detected with this MLPA probemix is between 95-98%, which is higher than in other populations. Only 51% of South African black SMA patients have a homozygous deletion of the SMN1 gene. The analytical sensitivity and specificity for the detection of deletions/duplications in the SMN1 and SMN2 genes (based on a 2006-2014 literature review) is very high and can be considered >99%.

Analytical performance can be compromised by: SNPs or other polymorphisms (e.g. indels) in the DNA target sequence, impurities in the DNA sample, incomplete DNA denaturation, the use of insufficient or too much sample DNA, the use of insufficient or unsuitable reference samples, problems with capillary

electrophoresis or a poor data normalisation procedure and other technical errors. The MLPA General Protocol contains technical guidelines and information on data evaluation/normalisation.

Data analysis: Coffalyser.Net software must be used for data analysis in combination with the appropriate lot-specific MLPA Coffalyser sheet. For both, the latest version should be used which are freely downloadable at www.mlpa.com. Use of other non-proprietary software may lead to inconclusive or false results. For more details on MLPA quality control and data analysis, see the Coffalyser.Net Manual.

Interpretation of results: The expected results for SMN1 and SMN2 specific MLPA probes are allele copy numbers of 2 (normal), 0 (homozygous deletion), 1 (heterozygous deletion), 3 and occasionally 4. Allele copy numbers of 3 or 4 are usually due to gene conversion.

The standard deviation of all probes in the reference samples should be <0.10 and the dosage quotient (DQ) of the reference probes in the patient samples should be between 0.80 and 1.20. When these criteria are fulfilled, the following cut-off values for the DQ of the probes can be used to interpret MLPA results:

**Copy Number status**	**Dosage quotient**
Normal	0.80 < DQ < 1.20
Homozygous deletion	DQ = 0
Heterozygous deletion	0.40 < DQ < 0.65
Heterozygous duplication	1.30 < DQ < 1.65
Heterozygous triplication/ Homozygous duplication	1.75 < DQ < 2.15
Ambiguous copy number	All other values

Table 1: Overview of expected results and the corresponding conclusions

**Finding**	**Conclusion**	**Explanation**
SMA symptoms<br>- SMN1 exon 7: 0 copies.<br>- SMN1 exon 8: 0 copies.	SMA patient	SMN1 is absent, as no copies of the distinct SMN1 exon 7 are present. The absence of both SMN1 exon 8 copies confirms this.
SMA symptoms<br>- SMN1 exon 7: 0 copies.<br>- SMN1 exon 8: 1 or more copies.	SMA patient	SMN1 is absent, as no copies of the determining SMN1 exon 7 sequence are found. Due to gene conversion, 1 or more copies of the characteristic SMN1 exon 8 sequence appear to have become incorporated in the SMN2 gene.
SMA symptoms<br>- SMN1 exon 7: 1 copy.	SMA patient	If the patient has SMA symptoms, but one copy of SMN1 exon 7 is present, the patient may belong to the group presenting compound heterozygosity. Sequencing might reveal a defect in the remaining SMN1 copy.
No SMA symptoms<br>- SMN1 exon 7: 1 copy.<br>- SMN1 exon 8: 1 copy.	SMA carrier	One copy of SMN1 is absent, making the person a carrier. The absence of one copy of the SMN1 exon 8 sequence confirms this.
No SMA symptoms<br>- SMN1 exon 7: 1 copy.<br>- A: SMN1 exon 8 copies > 1.<br>- B: SMN1 exon 8 copies = 0.	SMA carrier	One copy of SMN1 is absent, making the person a carrier. A: due to gene conversion, 1 (or more) copies of the characteristic SMN1 exon 8 have become incorporated in the SMN2 gene. B: an SMN2 exon 8 copy has replaced the characteristic SMN1 exon 8 copy.
No SMA symptoms<br>- 2 copies of SMN1 exon 7.	Most likely not a SMA carrier	Most likely this person is not a carrier. However, there is a possibility that both SMN1 copies lie on one chromosome. If there is a reason to believe that the person is a carrier (i.e. child is SMA-patient), he/she may belong to the 3-8% of carriers where this is indeed the case.

- Analysis of parental samples may be necessary for correct interpretation of complex results.
- False positive results: Please note that abnormalities detected by a single probe (or multiple consecutive probes) still have a considerable chance of being a false positive result. Abnormalities detected by a single probe may be due to a SNP very close to the ligation site. Sequence analysis can establish whether mutations or polymorphisms are present in the probe target sequence.

- False positive duplication results: Contamination of DNA samples with cDNA or PCR amplicons of individual exons can lead to false positive duplication results (Varga et al. 2012). Analysis of an independently collected secondary DNA sample can exclude these kinds of contamination artefacts.
- Copy number changes detected by reference probes are unlikely to have any relation to the condition tested for.

Notes SMA results**:**

SMA carrier screening:

- **False negative results:** The presence of two SMN1 exon 7 copies suggests that the person tested is not a carrier. However, this result can also be due to the presence of two SMN1 copies on one chromosome and 0 on the other, in which case the person tested is in fact a SMA carrier. Detection of some carriers is therefore compromised, as MLPA and other techniques are not able to identify carriers who have one chromosome lacking SMN1 with the other chromosome carrying two copies of SMN1**.**
- **False positive results:** Please note that individual MLPA probes can be affected differently by changes in experimental procedures or impurities in samples. Highly unlikely results such as an unusual high frequency of SMN1 exon 7 loss (carrier) or SMN1 exon 7 gain, without loss or gain of the exon 8 probe in most of these samples, should be treated with caution.

Limitations of the procedure:

- MLPA cannot detect any changes that lie outside the target sequence of the probes and will not detect copy number neutral inversions or translocations. Even when MLPA did not detect any aberrations, the possibility remains that biological changes in that gene or chromosomal region *do* exist but remain undetected.
- Sequence changes (e.g. SNPs, point mutations, small indels) in the target sequence detected by a probe can cause false positive results. Mutations/SNPs (even when >20 nt from the probe ligation site) can reduce the probe signal by preventing ligation of the probe oligonucleotides or by destabilising the binding of a probe oligonucleotide to the sample DNA.

Please report copy number changes detected by the reference probes, false positive results due to SNPs, and unusual results to MRC-Holland: info@mlpa.com.

Confirmation of results: As a way to confirm positive results, it is recommended to repeat the MLPA reaction with an independent DNA sample (independent DNA extraction). However, an apparent deletion detected by a single probe can be due to e.g. a mutation/polymorphism that prevents ligation or destabilises the binding of probe oligonucleotides to the DNA sample. Sequence analysis can establish whether mutations or polymorphisms are present in the probe target sequence.

Table 2. SALSA® MLPA® P060-B2 SMA probemix

Length (nt)	SALSA MLPA probe	Chromosomal position[(a)] reference	SMN1 / SMN2
64-70-76-82	Q-fragments (Only visible with <100 ng sample DNA)		
88-92-96	D-fragments (Low signal of 88 or 96 fragment indicates incomplete denaturation)		
100	X-fragment (X chromosome specific)		
105	Y-fragment (Y chromosome specific)		
154	Reference probe 02595-L17085	5q35	
163	Reference probe 02291-L17086	3p14	
172	Reference probe 02978-L17087	4q25	
**183**	**SMN1 probe** 14919-L17081		SMN1 exon 7
191	Reference probe 00559-L17088	11q22	
200	Reference probe 00976-L17298	11p13	
208	Reference probe 12490-L17096	1q32	
**218**	**SMN1 probe** 14881-L17082		SMN1 exon 8
228	Reference probe 14498-L17101	20p12	
237	Reference probe 02334-L17301	12q23	
245	Reference probe 14293-L17100	15q13	
255	Reference probe 13128-L17099	9q34	
264	Reference probe 07630-L17091	10q26	
272	Reference probe 14361-L17098	4q35	
**282**	**SMN2 probe** 14921-L17083		SMN2 exon 7
292	Reference probe 00824-L17097	3q25	
**301**	**SMN2 probe** 14878-L17084		SMN2 exon 8
311	Reference probe 06425-L17092	6p22	
321	Reference probe 01042-L17093	8q24	
331	Reference probe 01043-L17094	8q13	
342	Reference probe 13399-L17297	6q12	

(a) The exon numbering used in this P060-B2 SMA product description and in the P060-B2 SMA lot-specific Coffalyser.Net analysis sheet is the traditional exon numbering (exons 1, 2a, 2b, and 3-8). **Please note that the SMN1 and SMN2 exon numbering is different in the SMN1 and SMN2 LRG sequence and in the NCBI NG_008691.1 and NG_008728.1 reference sequence.**

Table 3. SMN1 and SMN2 probes in P060-B2

Length (nt)	SALSA MLPA probe	Gene Exon[(a)]	Partial sequence[(b)] (24 nt adjacent to ligation site)	Distance to next probe
183	14919-L17081	SMN1 exon 7	TTACAGGGTTTC-AGACAAAATCAA	0.7 kb
218	14881-L17082	SMN1 exon 8	GTAAAAGACTGG-GGTGGGGGTGGG	> 100 kb
282	14921-L17083	SMN2 exon 7	TTACAGGGTTTT-AGACAAAATCAA	0.8 kb
301	14878-L17084	SMN2 exon 8	GTAAAAGACTGA-GGTGGGGGTGGG	

(a) The exon numbering used in this P060-B2 SMA product description and in the P060-B2 SMA lot-specific Coffalyser.Net analysis sheet is the traditional exon numbering (exons 1, 2a, 2b, and 3-8). **Please note that the SMN1 and SMN2 exon numbering is different in the SMN1 and SMN2 LRG sequence and in the NCBI NG_008691.1 and NG_008728.1 reference sequence.**

(b) For visualisation purposes, the probe sequences are shown in forward orientation. Only partial sequences are shown. Complete probe sequences are available at www.mlpa.com. Please notify us of any mistakes: info@mlpa.com.

SALSA® MLPA® P060 SMA sample pictures

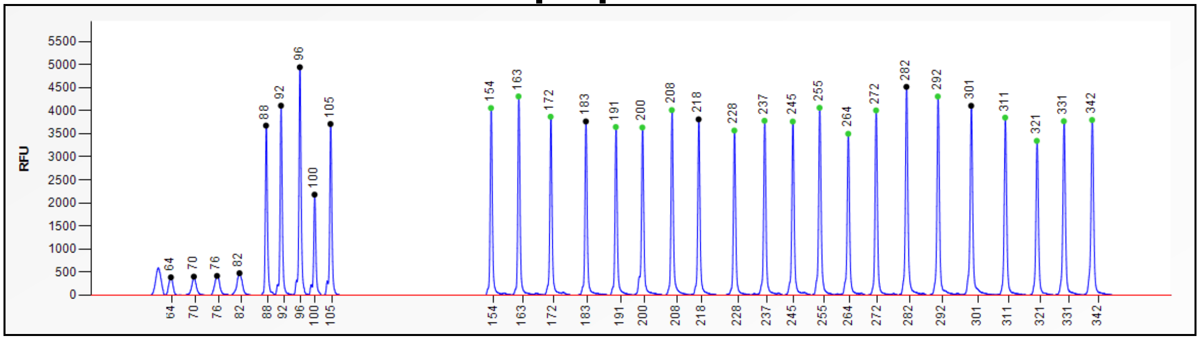

Figure 1. Capillary electrophoresis pattern of a sample of approximately 50 ng human male control DNA (2 copies each of SMN1 and SMN2) analysed with SALSA MLPA probemix P060 SMA (version B2).

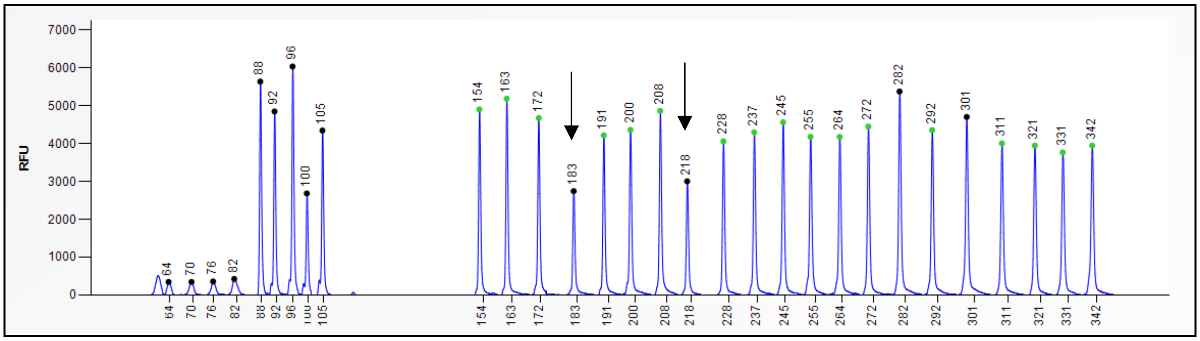

Figure 2. Capillary electrophoresis pattern of a sample of approximately 50 ng human male <u>SMA-carrier</u> DNA (1x SMN1, 2x SMN2) analysed with SALSA MLPA probemix P060 SMA (version B2).

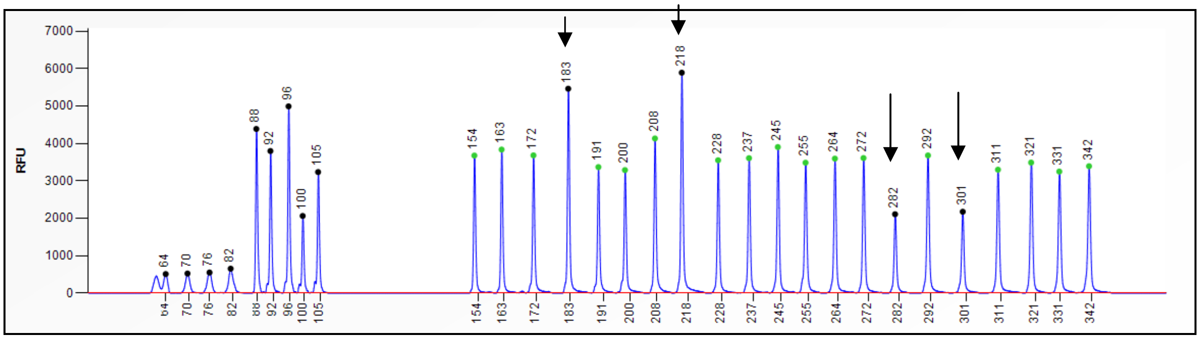

Figure 3. Capillary electrophoresis pattern of a sample of approximately 50 ng human male DNA (3x SMN1, 1x SMN2) analysed with SALSA MLPA probemix P060 SMA (version B2).

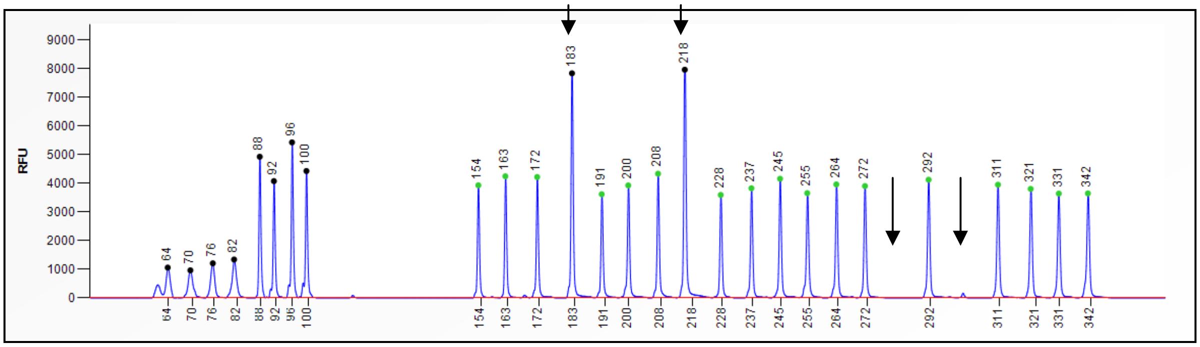

Figure 4. Capillary electrophoresis pattern of a sample of approximately 50 ng human female DNA (4x SMN1; 0x SMN2) analysed with SALSA MLPA probemix P060 SMA (version B2).

Related SALSA® MLPA® probemixes

P021 SMA	Spinal Muscular Atrophy (SMA), to determine SMN1 and SMN2 copy number changes (patients).
P058 IGHMBP2	Autosomal recessive distal spinal muscular atrophy 1 (DSMA1), contains probes for the IGHMBP2 gene.

References

- Alias L et al. (2014). Improving detection and genetic counseling in carriers of spinal muscular atrophy with two copies of the SMN1 gene. Clin Genet. 85:470-475.
- Arkblad EL et al. (2006). Multiplex ligation-dependent probe amplification improves diagnostics in spinal muscular atrophy. Neuromuscul Disord. 16:830-838.
- Ben-Shachar S et al. (2011). Large-scale population screening for spinal muscular atrophy: clinical implications. Genet Med. 13:110-114.
- Hendrickson BC et al. (2009). Differences in SMN1 allele frequencies among ethnic groups within North America. J Med Genet. 46:641-644.
- Labrum R et al. (2007). The molecular basis of spinal muscular atrophy (SMA) in South African black patients. Neuromuscul Disord. 17:684-692.
- Luo M et al. (2014). An Ashkenazi Jewish SMN1 haplotype specific to duplication alleles improves pan-ethnic carrier screening for spinal muscular atrophy. Genet Med. 16:149-156.
- Miskovic M et al. (2011). Lower incidence of deletions in the survival of motor neuron gene and the neuronal apoptosis inhibitory protein gene in children with spinal muscular atrophy from Serbia. Tohoku J Exp Med. 225:153-159.
- Schouten JP et al. (2002). Relative quantification of 40 nucleic acid sequences by multiplex ligation-dependent probe amplification. Nucleic Acids Res. 30:e57.
- Smith M et al. (2007). Population screening and cascade testing for carriers of SMA. Eur J Hum Genet. 15:759-766.
- Varga RE et al. (2012). MLPA-based evidence for sequence gain: pitfalls in confirmation and necessity for exclusion of false positives. Anal Biochem. 421:799-801.

Selected publications using SALSA MLPA Probemix P021/P060 SMA

- Alias L et al. (2011). Accuracy of marker analysis, quantitative real-time polymerase chain reaction, and multiple ligation-dependent probe amplification to determine SMN2 copy number in patients with spinal muscular atrophy. Genet Test Mol Biomarkers. 15:587-594.
- Amara A et al. (2012). Correlation of SMN2, NAIP, p44, H4F5 and Occludin genes copy number with spinal muscular atrophy phenotype in Tunisian patients. Eur J Paediatr Neurol. 16:167-174.
- CALÌ F et al. (2014). Carrier screening for spinal muscular atrophy in Italian population. J Genet. 93:179.
- Cao YY et al. (2013). [Detection of homozygous deletions in spinal muscular atrophy with genomic DNA sequencing]. Zhonghua Yi Xue Yi Chuan Xue Za Zhi. 30:410-414.
- DING Y et al. (2012). Application of muhiplex ligation-dependent probe amplification in molecular diagnosis of spinal muscular atrophy. Journal of Clinical Pediatrics. 11:002.
- Eggermann T et al. (2008). A new splice site mutation in the SMN1 gene causes discrepant results in SMN1 deletion screening approaches. Neuromuscul Disord. 18:146-149.
- He J et al. (2013). Molecular analysis of SMN1, SMN2, NAIP, GTF2H2, and H4F5 genes in 157 Chinese patients with spinal muscular atrophy. Gene. 518:325-329.
- Huang C-H et al. (2007). Copy number analysis of survival motor neuron genes by multiplex ligation-dependent probe amplification. Genet Med. 9:241-248.
- Jin YW et al. (2012). [Limitation of PCR-RFLP method for the detection of genetic mutations in spinal muscular atrophy]. Zhonghua Yi Xue Yi Chuan Xue Za Zhi. 29:34-37.
- Kang SH et al. (2009). False homozygous deletions of SMN1 exon 7 using Dra I PCR-RFLP caused by a novel mutation in spinal muscular atrophy. Genet Test Mol Biomarkers. 13:511-513.
- Kim J et al. (2010). Association between survivor motor neuron 2 (SMN2) gene homozygous deletion and sporadic lower motor neuron disease in a Korean population. Ann Clin Lab Sci. 40:368-374.
- Landaburu I et al. (2013). Genetic testing of sperm donors for cystic fibrosis and spinal muscular atrophy: evaluation of clinical utility. Eur J Obstet Gynecol Reprod Biol. 170:183-187.
- Lee J-B et al. (2012). Homozygous SMN2 deletion is a major risk factor among twenty-five Korean sporadic amyotrophic lateral sclerosis patients. Yonsei Med J. 53:53-57.

- Miskovic M et al. (2014). Ten years of experience in molecular prenatal diagnosis and carrier testing for spinal muscular atrophy among families from Serbia. Int J Gynaecol Obstet. 124:55-58.
- Najmabadi H et al. (2009). Quantitative analysis of SMN1 gene and estimation of SMN1 deletion carrier frequency in Iranian population based on real-time PCR. Genetics in the 3rd millennium. 7:1760-1760.
- Strom CM et al. (2013). 1000 sample comparison of MLPA and RT-PCR for carrier detection and diagnostic testing for Spinal Muscular Atrophy Type 1. Open J Genet. 3:111.
- Stuppia L et al. (2012). Use of the MLPA assay in the molecular diagnosis of gene copy number alterations in human genetic diseases. Int J Mol Sci. 13:3245-3276.
- Tomaszewicz K et al. (2005). Detection of homozygous and heterozygous SMN deletions of spinal muscular atrophy in a single assay with multiplex ligation-dependent probe amplification. Beijing Da Xue Xue Bao. 37:55-57.
- WANG J et al. (2013). Copy number variation of SMN1 and SMN2 genes in spinal muscular atrophy and analysis of its clinical significance. Chinese Journal of Evidence-Based Pediatrics. 3:025.
- Yan G et al. (2010). Gene diagnosis and carriers detection of spinal muscular atrophy by multiplex ligation-dependent probe amplification. Chinese Journal of Clinicians. 4:1512-1519.
- Yoon S et al. (2010). Determination of SMN1 and SMN2 copy numbers in a Korean population using multiplex ligation-dependent probe amplification. Korean J Lab Med. 30:93-96.
- Yu-Jin Q et al. (2012). Subtle mutations in the SMN1 gene in Chinese patients with SMA: p.Arg288Met mutation causing SMN1 transcript exclusion of exon7. BMC Med Genet. 13:86.
- Yupeng W et al. (2008). Gene Diagnosis of Spinal Muscular Atrophy Using MLPA and PCR-RFLP. Chinese Journal of Family Planning. 9:008.
- Zapletalova E et al. (2007). Analysis of point mutations in the SMN1 gene in SMA patients bearing a single SMN1 copy. Neuromuscul Disord. 17:476-481.
- Zeng J et al. (2008). Evaluation of an in-house protocol for prenatal molecular diagnosis of SMA in Chinese. Clin Chim Acta. 398:78-81.
- Zeng J et al. (2011). Establishment of a molecular diagnostic system for spinal muscular atrophy experience from a clinical laboratory in china. J Mol Diagn. 13:41-47.
- Zhu H et al. (2010). [Studies on the molecular diagnosis and prenatal diagnosis of the spinal muscular atrophy carriers by multiplex ligation-dependent probe]. Zhonghua Yi Xue Yi Chuan Xue Za Zhi. 27:38-41.

**P060 Product history**	
Version	*Modification*
B2	The 88 and 96 nt DNA denaturation control fragments have been replaced (QDX2).
B1	Completely redesigned product. SMN2 exon 7 and 8 probes are now included.
A2	Two extra control fragments at 100 and 105 nt, specific for chromosome X and Y, have been added.
A1	First release.

**Implemented changes in the product description**
Version B2-04 – 07 October 2016 (03)<br>- Intended use and required specimens sections updated.<br>*Version B2-03 – 02 August 2016 (03)*<br>- Product description adapted to a new template.<br>- Intended use and required specimens sections updated.<br>*Version B2-02 – 24 March 2016 (02)*<br>- Remark added under Table 3 about probe orientation.<br>*Version B2-01 – 21 July 2015 (02)*<br>- Product description completely rewritten and adapted to new template.<br>*Version 24 – 10 March 2015 (54)*<br>- New sample picture included in product description.<br>- Data analysis method has been modified.<br>*Version 20 – 06 August 2013 (50)*<br>- Product description adapted to a new lot (lot number added, new picture included).<br>*Version 19 – 01 December 2012 (48)*<br>- Product description adapted to a new lot (lot number added, new picture included).<br>*Version 18 – 05 October 2012 (48)*

- Remark on exon numbering added below Table 3.<br>- Electropherogram pictures using the new MLPA buffer (introduced in December 2012) added.

**More information: www.mlpa.com; www.mlpa.eu**	
	MRC-Holland bv; Willem Schoutenstraat 1<br>1057 DL, Amsterdam, The Netherlands
E-mail	info@mlpa.com (information & technical questions); order@mlpa.com (orders)
Phone	+31 888 657 200

***EUROPE:** CE IVD

OUTSIDE EUROPE: RUO

*comprising EU member states, EU member state candidates and members of the European Free Trade Association (EFTA).
The product is for RUO in all other countries within Europe
