# Supplementary material for: Newborn Screening for Spinal Muscular Atrophy in China Using DNA Mass Spectrometry
Source: Front Genet. 2019 Dec 17;10:1255. doi: 10.3389/fgene.2019.01255 (PMC6928056; doi:10.3389/fgene.2019.01255)
Supplement: Supplementary file 3 [file DataSheet_3.pdf]

MLPA® General Protocol

Instructions For Use

MLPA (Multiplex Ligation-dependent Probe Amplification)
General Protocol for the detection and quantification of DNA sequences.

This protocol contains information that is essential for obtaining reliable MLPA results. It must be read in its entirety and used in combination with the appropriate MLPA probemix-specific product description.

SALSA® MLPA® reagent kits and Coffalyser.Net analysis software are registered for in vitro diagnostic use (IVD) in specific countries (see www.mrcholland.com). In all other countries, these products are for research use only (RUO). When using an IVD-registered probemix for diagnostic purposes, it is essential to combine it with SALSA® MLPA® reagent kits and Coffalyser.Net analysis software. Country-specific information on the IVD status of probemixes can be found in the appropriate probemix-specific product description and at www.mrcholland.com.

A separate protocol exists for the detection of DNA copy number and methylation status (MS-MLPA®). This protocol is available at www.mrcholland.com.

Manufacturer: MRC Holland B.V. Willem Schoutenstraat 1, 1057 DL Amsterdam, The Netherlands
Website: www.mrcholland.com; Phone: +31 888 657 200
E-mail: info@mrcholland.com (information & technical questions), order@mrcholland.com (orders)

Table of Contents

1. INTRODUCTION 2
 1.1. SALSA MLPA ASSAY COMPONENTS & STORAGE CONDITIONS 2
 1.1.1. REAGENT KIT ITEM NUMBERS 2
 1.1.2. REAGENT KIT COMPONENTS 3
 1.1.3. APPLICATION-SPECIFIC MLPA PROBEMIX 3
 1.1.4. STORAGE AND SHELF LIFE 3
 1.1.5. PACKAGING LABELS 3
 1.2. MLPA ASSAY PRINCIPLE 3
2. ASSAY SETUP INSTRUCTIONS 4
 2.1. MATERIALS REQUIRED BUT NOT PROVIDED 4
 2.2. SAMPLE TREATMENT AND STORAGE 5
 2.3. SELECTING REFERENCE & OTHER CONTROL SAMPLES 5
3. NOTES TO READ BEFORE YOU START 6
4. CRITICAL POINTS FOR OBTAINING GOOD MLPA RESULTS 6
5. MLPA PROTOCOL - IN BRIEF 6
6. MLPA PROTOCOL 7
 6.1. THERMOCYCLER PROGRAM FOR THE MLPA REACTION 7
 6.2. DNA DENATURATION (DAY 1) 7
 6.3. HYBRIDISATION REACTION (DAY 1) 7
 6.4. LIGATION REACTION (DAY 2) 7
 6.5. PCR REACTION (DAY 2) 7
7. FRAGMENT SEPARATION BY CAPILLARY ELECTROPHORESIS 7
 7.1. NOTES TO READ BEFORE YOU START 7
 7.2. ELECTROPHORESIS SPECIFICATIONS 8
8. MLPA QUALITY CONTROL AND TROUBLESHOOTING 8
 8.1. MLPA QUALITY CONTROL FRAGMENTS 8
 8.2. NO-DNA CONTROL 9
 8.3. EVAPORATION 9
 8.4. QUALITY CONTROL FLOWCHART 10
9. DATA ANALYSIS 11
10. INTERPRETATION AND CONFIRMATION 11
11. PRECAUTIONS AND WARNINGS 11
12. LIMITATIONS OF THE PROCEDURE 11

1. INTRODUCTION

Copy number variations (CNVs) are a prominent source of genetic variation in human DNA and play a role in a large number of disorders. Multiplex Ligation-dependent Probe Amplification (MLPA®) is a semi-quantitative, non-automated technique that is used to determine the relative copy number of up to 60 DNA sequences in a single multiplex PCR-based reaction.

1.1. SALSA MLPA ASSAY COMPONENTS & STORAGE CONDITIONS

1.1.1. REAGENT KIT ITEM NUMBERS

Cat No	Description	Number of reactions	Fluorescent label PCR primer
EK1-FAM or EK1-Cy5	SALSA MLPA EK1 reagent kit	100	FAM or Cy5
EK5-FAM or EK5-Cy5	SALSA MLPA EK5 reagent kit	500	FAM or Cy5
EK20-FAM	SALSA MLPA EK20 reagent kit	2000	FAM

1.1.2. REAGENT KIT COMPONENTS

Reagent kit component	Volumes			Ingredients[1]
	EK1	EK5	EK20	
SALSA MLPA Buffer (yellow cap)	180 µl	5×180 µl	5×700 µl	KCl, Tris-HCl, EDTA, PEG-6000, DTT, oligonucleotides
SALSA Ligase-65 (green cap)	115 µl	5×115 µl	5×460 µl	Glycerol, EDTA, DTT, KCl, Tris-HCl, non-ionic detergent, Ligase-65 enzyme (bacterial origin)
SALSA Ligase Buffer A (transparent cap)	360 µl	5×360 µl	5×1420 µl	Coenzyme NAD (bacterial origin)
SALSA Ligase Buffer B (white cap)	360 µl	5×360 µl	5×1420 µl	Tris-HCl, $MgCl_2$, non-ionic detergent
SALSA PCR Primer Mix (brown cap)	240 µl	5×240 µl	5×940 µl	Synthetic oligonucleotides with fluorescent dye (FAM or Cy5), dNTPs, Tris-HCl, KCl, EDTA, non-ionic detergent
SALSA Polymerase (orange cap)	65 µl	5×65 µl	5×240 µl	Glycerol, non-ionic detergents, EDTA, DTT, KCl, Tris-HCl, Polymerase enzyme (bacterial origin)

1.1.3. APPLICATION-SPECIFIC MLPA PROBEMIX

Application-specific MLPA probemix	Available Volumes (R=number of reactions)	Ingredients
Probemix* (black cap)	40 µl (25R), 80 µl (50R), 160 µl (100R)	Synthetic oligonucleotides, oligonucleotides purified from bacteria, Tris-HCl, EDTA
Sample DNA# (SD) (blue cap)	30 µl or 100 µl	Tris-HCl, EDTA, synthetic/control plasmid DNA, human genomic female DNA, cell line DNA

* Probemixes are designed for use only in combination with SALSA MLPA reagent kits.
# A vial of SD (reference (selection), binning, or artificial duplication DNA) is supplied with or can be separately ordered for certain MLPA probemixes. Volumes and ingredients are dependent on SD type.

1.1.4. STORAGE AND SHELF LIFE

All components must be stored directly upon arrival, and after use, between -25°C and -15°C, shielded from light and in the original packaging. When stored under the recommended conditions, a shelf life of until the expiry date is guaranteed, also after opening. For the exact expiry date, see the labels on each vial. Products should not be exposed to more than 25 freeze-thaw cycles.

1.1.5. PACKAGING LABELS

	Manufacturer		Store at
LOT	Lot Number		Keep away from heat or direct sunlight
	Use by	REF	Catalogue Number
Σ	Number of Tests	i	Read instructions before use
IVD	In Vitro Diagnostic	RUO	Research Use Only

1.2. MLPA ASSAY PRINCIPLE

The principle of MLPA is based on the amplification of up to 60 probes that each detect a specific DNA sequence of approximately 60 nt in length (Figure 1)[2]. The MLPA reaction results in a set of unique PCR amplicons between 64-500 nt in length that are separated by capillary electrophoresis. After initial denaturation of the sample DNA, a mixture of MLPA probes is added to the sample. In general, each MLPA probe consists of two oligonucleotides that

[1] None of the ingredients are derived from humans, animals, or pathogenic bacteria. Based on the concentrations present, none of the ingredients are hazardous as defined by the Hazard Communication Standard. **A Safety Data Sheet (SDS) is not required for these products:** none of the preparations contain dangerous substances (as per Regulation (EC) No 1272/2008 [EU-GHS/CLP] and amendments) at concentrations requiring distribution of an SDS (as per Regulation (EC) No 1272/2008 [EU-GHS/CLP] and 1907/2006 [REACH] and amendments). If spills occur, clean with water and follow appropriate site procedures.
[2] Schouten JP et al. (2002). Relative quantification of 40 nucleic acid sequences by multiplex ligation-dependent probe amplification. *Nucleic Acids Res.* 30:e57.

must hybridise to directly adjacent target sequences in order to be ligated into a single probe (Figure 1). During the subsequent PCR reaction, all ligated probes are amplified simultaneously using the same PCR primer pair, resulting in a set of unique PCR amplicons. One PCR primer is fluorescently labelled, enabling the amplification products to be visualised during fragment separation on a capillary electrophoresis instrument. Fragment separation yields a sample-specific electropherogram: the sample peak pattern (Figure 2, top).

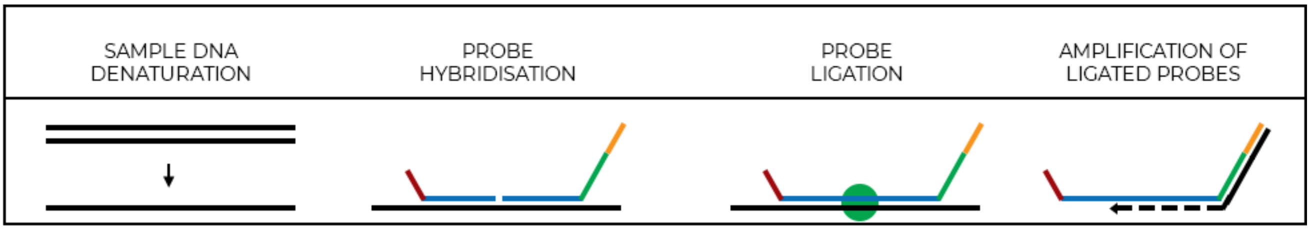

Figure 1. MLPA reaction.

MLPA is a relative technique: only relative differences can be detected by comparing the MLPA peak patterns of DNA samples. The relative height of each individual probe peak, as compared to the relative probe peak heights in various reference DNA samples, reflects the relative copy number of the corresponding target sequence in a sample. Inclusion of reference samples in the same run is therefore essential. A deletion of one or more target sequences is visible as a relative decrease in peak height (Figure 2, bottom), while an increase in relative peak height reflects a copy number increase.

Figure 2. Profile comparison of MLPA data.

Top: Electropherogram of test sample A (right) is compared to those of the reference samples (left). A relative decrease of two probes is seen in test sample A (circled in red).

Bottom: Calculated probe ratios of test sample A (right) normalised to the reference samples (left), as displayed by Coffalyser.Net software. Arranging probes by chromosomal location reveals a heterozygous deletion, probe ratio 0.5, in the test sample (red dots).

T: target probes, R: reference probes.

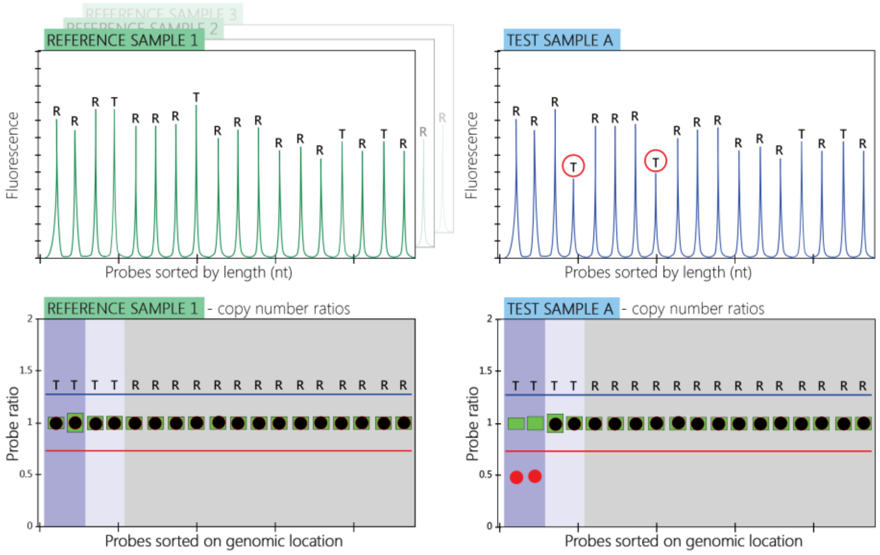

2. ASSAY SETUP INSTRUCTIONS

2.1. MATERIALS REQUIRED BUT NOT PROVIDED

- Ultrapure water
- $TE_{0.1}$ (10 mM Tris-HCl pH 8.0 + 0.1 mM EDTA)
- Calibrated thermocycler with heated lid (99-105°C) and standard laboratory equipment
- 0.2 ml PCR tubes, strips or plates
- Capillary electrophoresis instrument[3] with fragment analysis software
  - Applied Biosystems: Standard Foundation Data Collection Software
  - SCIEX: GeXP Software Package
- High quality formamide (e.g. Hi-Di Formamide, Applied Biosystems)
- Labelled size standard
  - Applied Biosystems: GeneScan™ 500 LIZ®/ROX™ (preferred; mandatory for use with IVD-registered probemixes), GeneScan™ 600 LIZ®, GeneScan™ 500 TAMRA™
  - SCIEX: CEQ™ DNA Size Standard Kit - 600

[3] Capillary electrophoresis instruments that do not use denaturing conditions, like QIAGEN QIAxcel or Agilent Fragment Analyzer, cannot be used in combination with MLPA.

- Polymers
  - Applied Biosystems: POP-4 or POP-7 are preferred. POP-6 is not recommended due to its high resolution. SeqStudio: POP-1 is integrated in the cartridge and is suitable.
  - SCIEX: GenomeLab™ Linear Polyacrylamide (LPA) denaturing gel
- Coffalyser.Net analysis software (freely downloadable at www.mrcholland.com)

2.2. SAMPLE TREATMENT AND STORAGE

- Use a total quantity of 50-250 ng of human DNA (50-100 ng is optimal; unless stated otherwise in the probemix-specific product description) in a 5 µl[4] volume for each MLPA reaction[5]. If necessary, DNA samples can be concentrated by ethanol precipitation, and glycogen (Roche 901393) can be used as a carrier. For more information visit www.mrcholland.com.
- DNA preparations should contain 5-10 mM Tris buffer with a pH of 8.0-8.5 to prevent depurination during the initial denaturation step at 98°C. For example, dissolve and dilute sample DNA in **$TE_{0.1}$** (10 mM Tris-HCl pH 8.0 + 0.1 mM EDTA). If it is unknown whether sufficient buffering capacity is present, add Tris-HCl: 4 µl sample DNA + 1 µl 50 mM Tris-HCl pH 8.5.
- Contaminants remaining after DNA extraction, including NaCl or KCl (>40 mM) and other salts, phenol, ethanol, heparin, EDTA (>1.5 mM) and Fe, may influence MLPA performance. MLPA is more sensitive to impurities than monoplex PCR assays. Do not concentrate DNA by evaporation or SpeedVac; this leads to high EDTA and salt concentrations.
- Ensure that the extraction method, tissue type, DNA concentration and treatment are as similar as possible in test and reference samples.
- Extraction methods should not leave a high concentration of contaminants. Do not use QIAGEN M6, M48 and M96 systems, as they leave too much salt. For QIAGEN EZ1, use the *QIAGEN Supplementary Protocol: Purification of genomic DNA from whole blood, optimized for use in MRC-Holland MLPA® assays, using EZ1® DNA Blood Kits* (see www.mrcholland.com). MRC Holland has tested and can recommend the following extraction methods:
  - QIAGEN Autopure LS (automated) and QIAamp DNA mini/midi/maxi kit (manual)
  - Promega Wizard Genomic DNA Purification Kit (manual)
  - Salting out (manual)
- Heparinised blood can only be used when the sample has undergone a purification method to remove the heparin contamination (e.g. Nucleospin gDNA Clean-up XS).
- An RNAse treatment is only essential when examining genes that are highly expressed in the sample tissue studied. E.g. *HBA* and *HBB* in blood-derived samples; (mitochondrial) ribosomal RNA genes (all tissues).
- In certain cases, the SALSA Sample Stabilising Solution (S4; Cat No SMR04, SMR45) (RUO) can improve the quality of the MLPA reaction. See the product description at www.mrcholland.com for more information.
- DNA from whole genome amplification reactions (WGA) is not suitable for MLPA due to amplification bias.
- Aliquot samples and store at -20°C. Contamination with microorganisms can deteriorate samples that are stored at 4°C for an extended period.

2.3. SELECTING REFERENCE & OTHER CONTROL SAMPLES

- SELECTING REFERENCE SAMPLES. Reference samples are DNA samples obtained from healthy individuals with a normal copy number for the sequences detected by the target and reference probes. They should be as similar as possible to test samples in all other aspects, including extraction method and sample source. Please note that not all probemixes are suitable for use with DNA from all sources (e.g. formalin-fixed paraffin-embedded (FFPE) tissue). Always check probemix product descriptions for suitable DNA sources.
- REFERENCE SAMPLES. At least three reference samples should be included in each MLPA experiment. When testing >21 samples, include one additional reference sample per seven additional test samples. Distribute reference samples randomly over the experiment to minimise variation. **Multiple reference samples** are needed to estimate the reproducibility of each probe within each MLPA run.
- COMMERCIAL DNA. In case of doubts about sample quality, include one or more commercial DNA samples for comparison. We recommend Promega Cat No G1471 male & G1521 female DNA. The commercial DNA should only be used as a control to check sample quality and cannot be used as a reference sample.
- NO-DNA CONTROL. It is recommended to include a no-DNA control in every MLPA run. Replace 5 µl DNA by $TE_{0.1}$ (10 mM Tris-HCl pH 8.0 + 0.1 mM EDTA) to check for contamination of TE, MLPA reagents, electrophoresis reagents or capillaries.

---

[4] Never use more than 5 µl sample DNA per reaction. Using more than 5 µl DNA reduces the probe and salt concentration. This reduces the hybridisation speed and the stability of the binding of MLPA probes to the sample DNA.

[5] Optical density (260 nm) measurements often overestimate the DNA concentration, e.g. due to contamination with RNA. Whether the DNA quantity was sufficient can be estimated on the basis of the Q-fragments, as is explained in 8.1.

- POSITIVE CONTROL SAMPLES. Inclusion of positive control samples is recommended when available. MRC Holland does not provide positive samples, but a list of commercially available positive samples is available on www.mrcholland.com. When using cell line DNA, note that cell lines may have acquired additional copy number changes, including gains or losses of complete chromosomes.

3. NOTES TO READ BEFORE YOU START

- Always vortex thawed buffers and probemix, followed by brief centrifugation. All enzyme reagent tubes should be centrifuged briefly. MLPA buffer is typically frozen at -20°C but may remain liquid due to its high salt concentration.
- Before use, warm enzyme vials (Ligase-65 and polymerase) for 10 sec in your hand to reduce viscosity.
- Enzyme solutions contain 50% glycerol and remain liquid at -20°C. Master mixes containing enzymes should be mixed thoroughly by gently pipetting up and down. Insufficient mixing can result in unreliable results. When preparing master mixes, always add enzymes last. **Never vortex solutions containing enzymes** as enzyme inactivation can occur.
- To minimise sample variation, prepare sufficiently large volumes of master mix solutions (5-10% volume surplus).
- Prepare master mixes (Ligase-65 and polymerase) at room temperature (RT) right before use. When prepared >1 hr before use, store master mixes on ice or at 4°C. Master mixes should be warmed to RT before addition to MLPA reactions. Non-specific peaks may form in the no-DNA reaction when very cold ligase master mix is added.
- Use multichannel pipettes to avoid excessive evaporation.
- A video on how to perform an MLPA reaction in the lab can be found on www.mrcholland.com.

4. CRITICAL POINTS FOR OBTAINING GOOD MLPA RESULTS

- Ensure all DNA samples contain 5-10 mM Tris-HCl pH 8-8.5. (Section 2.2)
- Include at least three reference samples, derived from the same tissue type and treated in the same manner as test samples, in every MLPA experiment. (Section 2.3)
- Accurate pipetting of reagents is essential to obtain reliable results. This is especially critical for the 3 µl of hybridisation master mix. (Section 6)
- Use Coffalyser.Net for data analysis. (Section 9)
- Check quality fragments. Complete sample DNA denaturation is essential. (Section 8)
- Perform regular CE device maintenance, replacing capillaries and polymer as recommended by the manufacturer. (Section 7)

5. MLPA PROTOCOL - IN BRIEF

1. DNA DENATURATION
   - Heat a 5 µl DNA sample for 5 minutes at 98°C
2. HYBRIDISATION OF PROBES TO SAMPLE DNA
   - Cool down to room temperature, open tubes
   - Add 3 µl hybridisation master mix*
   - Incubate 1 minute at 95°C and hybridise for 16 hours at 60°C
3. LIGATION OF HYBRIDISED PROBES
   - Lower thermocycler temperature to 54°C, open tubes
   - Add 32 µl Ligase-65 master mix*, incubate 15 minutes at 54°C
   - Heat inactivate the ligase enzyme: 5 minutes 98°C
4. PCR AMPLIFICATION OF LIGATED PROBES
   - Cool down to room temperature, open tubes
   - Add 10 µl polymerase master mix* at room temperature
   - Start PCR (35 x {95°C 30 seconds, 60°C 30 seconds, 72°C 60 seconds}, 72°C 20 minutes, 15°C pause)
5. FRAGMENT SEPARATION BY CAPILLARY ELECTROPHORESIS
6. ANALYSE RESULTS WITH COFFALYSER.NET

* Master mixes:
- Hybridisation: 1.5 µl SALSA probemix +1.5 µl MLPA buffer, per reaction
- Ligase-65: 3 µl ligase buffer A + 3 µl ligase buffer B + 25 µl ultrapure water + 1 µl Ligase-65, per reaction
- Polymerase: 7.5 µl ultrapure water + 2 µl PCR primer mix + 0.5 µl SALSA polymerase, per reaction

6. MLPA PROTOCOL

6.1. THERMOCYCLER PROGRAM FOR THE MLPA REACTION

DNA denaturation				
1.	98°C	*5 minutes*		
2.	25°C	*pause*		
Hybridisation reaction				
3.	95°C	*1 minute*		
4.	60°C	*16-20 hours*		
Ligation reaction				
5.	54°C	*pause*		
6.	54°C	*15 minutes*		
7.	98°C	*5 minutes*		
8.	20°C	*pause*		
PCR reaction				
9.	35 cycles:	•	95°C	*30 seconds*
		•	60°C	*30 seconds*
		•	72°C	*60 seconds*
10.	72°C	*20 minutes*		
11.	15°C	*pause*		

Note: This thermocycler program should be followed unless stated otherwise in the probemix-specific product description.

6.2. DNA DENATURATION (DAY 1)

- Label 0.2 ml tubes, strips or plates.
- Add 5 µl DNA sample (50-250 ng; 50-100 ng is optimal) or TE (no-DNA control) to each tube.
- Place tubes in thermocycler; start MLPA thermocycler program steps 1-2 (see section 6.1).
- Ensure samples are at 25°C before removing tubes from the thermocycler.

6.3. HYBRIDISATION REACTION (DAY 1)

- Prepare hybridisation master mix. For each reaction, mix: 1.5 µl MLPA buffer (yellow cap) + 1.5 µl probemix (black cap). Mix well by pipetting or vortexing.
- After DNA denaturation, add 3 µl hybridisation master mix to each reaction. Accurate pipetting is critical. Mix well by pipetting gently up and down.
- Continue thermocycler program with steps 3-4.

6.4. LIGATION REACTION (DAY 2)

- Prepare a Ligase-65 master mix. For each reaction, mix: 25 µl ultrapure water + 3 µl ligase buffer A (transparent cap) + 3 µl ligase buffer B (white cap), then add 1 µl Ligase-65 enzyme (green cap). Mix well by pipetting gently up and down.
- Continue thermocycler program with step 5.
- When the thermocycler is at 54°C and **while the samples are IN the thermocycler**, add 32 µl Ligase-65 master mix to each MLPA reaction. Mix well by pipetting gently up and down.
- Continue thermocycler program with steps 6-8.

6.5. PCR REACTION (DAY 2)

- Prepare polymerase master mix. For each reaction, mix: 7.5 µl ultrapure water + 2 µl SALSA PCR primer mix (brown cap), then add 0.5 µl SALSA polymerase (orange cap). Mix well by pipetting gently up and down.
- **At room temperature**, add 10 µl polymerase master mix to each MLPA reaction. Mix well by pipetting gently up and down. **Immediately** place the tubes in the thermocycler and continue the thermocycler program with steps 9-11.
- After the PCR reaction, do <u>not</u> open tubes in the same room as the thermocycler. To avoid contamination, use different micropipettes for performing MLPA reactions and handling MLPA PCR products.
- PCR product can be stored shielded from light at 4°C for 1 week. For longer periods, store between -25°C and -15°C.

7. FRAGMENT SEPARATION BY CAPILLARY ELECTROPHORESIS

7.1. NOTES TO READ BEFORE YOU START

- Size standard, run conditions, polymer, fluorescent dye and volume of MLPA PCR reaction depend on the capillary electrophoresis instrument type. Use the default fragment analysis settings on your capillary

electrophoresis instrument applicable for the application, polymer and capillary length. Instrument settings may require optimisation for proper fragment separation.
- Replace capillaries and polymer regularly, by following manufacturer recommendations. Polymer quickly deteriorates after prolonged exposure to >25°C. If size standard peaks are repeatedly low and broad, the capillaries or polymer may have deteriorated.
- Use high quality formamide and store it in aliquots at -20°C. Formamide can become acidic, causing depurination and fragmentation of DNA upon heating.

7.2. ELECTROPHORESIS SPECIFICATIONS

Instrument	Primer Dye	Capillaries	Injection mixture
SCIEX<br>CEQ-2000<br>CEQ-8000<br>CEQ-8800<br>GeXP	Cy5	33 cm	1 µl PCR reaction[a]<br>0.5 µl CEQ - size standard 600[b]<br>28.5 µl HiDi formamide / Beckman SLS<br>Add one drop of high quality mineral oil.
ABI-Prism 3100 (Avant)<br>ABI-3130 (xL)<br>ABI-3500[c] (xL)<br>ABI-3730 (xL)	FAM	36, 50 cm	0.7 µl PCR reaction[a]<br>0.3 µl ROX or 0.2 µl LIZ GS 500 size standard<br>9 µl HiDi formamide<br>Seal the injection plate. Heat 3 min at 86°C, cool for 2 min at 4°C[d].
ABI-SeqStudio	FAM	28 cm	0.8 µl PCR reaction[a]<br>0.3 µl ROX/LIZ GS500 size standard<br>12 µl HiDi formamide<br>Seal the injection plate. Heat 3 min at 86°C, cool for 2 min at 4°C[d].

[a] The volume of PCR product added should never exceed 10% of the total injection mixture.
[b] Reduce volume of size standard if needed.
[c] For ABI-3500: set run voltage to 15 kV and ensure sufficient run time.
[d] Briefly heating the injection mixture before capillary electrophoresis is recommended.

The table below contains the optimal, minimum, and maximum signal ranges for the capillary electrophoresis instruments. If signals falls outside of these values, false results can be obtained. Optimisation of the fragment analysis settings may be required.

Instrument	Optimal signal range (in RFU)	Minimum signal (in RFU)	Maximum signal (in RFU)
SCIEX CEQ/GeXP	9,375 - 136,000	5000	170,000
ABI 310, 3100 & 3130 series	375 - 6,000	200	7,500
ABI 3500, 3730 series & SeqStudio	375 - 24,800	300	31,000

8. MLPA QUALITY CONTROL AND TROUBLESHOOTING

8.1. MLPA QUALITY CONTROL FRAGMENTS

Coffalyser.Net should be used for MLPA data analysis as it automatically performs control fragment checks to ensure minimal quality requirements are met! MLPA probemixes contain quality control fragments that signal if there are problems that may affect MLPA results. Evaluate the quality of the MLPA reaction, including quality control fragments, using the quality control flowchart (section 8.4). Only data that meets the quality requirements is suitable for MLPA result interpretation. To aid in quality assessment, the e-learning modules MLPA quality control fragments and MLPA troubleshooting wizard are available online at www.mrcholland.com.

Almost all SALSA MLPA probemixes contain nine control fragments, as described below:

Name	Length (nt)	Interpretation
Benchmark fragment	92	Benchmark to compare other quality control fragments to.
Quantity fragments (Q-fragments)	64, 70, 76, 82	**High** when DNA amount is too low or ligation failed. Median of Q-fragment signals ≥33% of the 92 nt benchmark fragment → DNA quantity insufficient or ligation failed. See Figure 3.
Denaturation fragments (D-fragments)	88, 96	**Low** in case of poor sample DNA denaturation. Signal ≤50% of the 92 nt benchmark fragment → DNA denaturation insufficient. See Figure 4.
X & Y fragments	100, 105	Control for sample mix up[6].

[6] Cases are known of males lacking this Y-specific sequence and females carrying this Y-sequence on an X-chromosome.

Q-FRAGMENTS

The four Q-fragments (64, 70, 76 & 82 nt) provide a control for sufficient DNA addition and successful ligation. The Q-fragments do not need to hybridise to the DNA or be ligated in order to be amplified during PCR. The Q-fragments decrease in height as more sample DNA is included in a reaction (Figure 3).

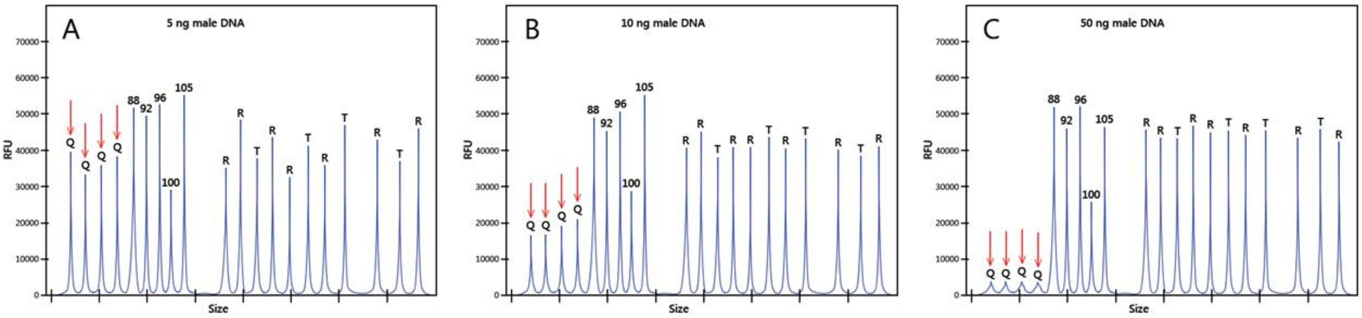

Figure 3. Effect of DNA quantity on Q-fragments. The more sample DNA is used, the lower the Q-fragments. MLPA results with **A.** 5 ng, **B.** 10 ng, **C.** 50 ng DNA. Samples **A.** and **B.** contain insufficient DNA.

D-FRAGMENTS

The two D-fragments (88 & 96 nt) detect sequences in exceptionally strong CpG islands. CpG islands have a high GC-content and are difficult to denature. When the 88 and 96 nt D-fragments are low ($\leq$50% of the 92 nt benchmark fragment) this indicates sample DNA was insufficiently denatured. Poor denaturation can be due to the presence of >40 mM salt in a DNA sample. Incomplete sample DNA denaturation can result in false results!

NOTE: When using ABI POP7 polymer, a non-specific fragment of 80-90 nt is usually present that may coincide with the control fragments!

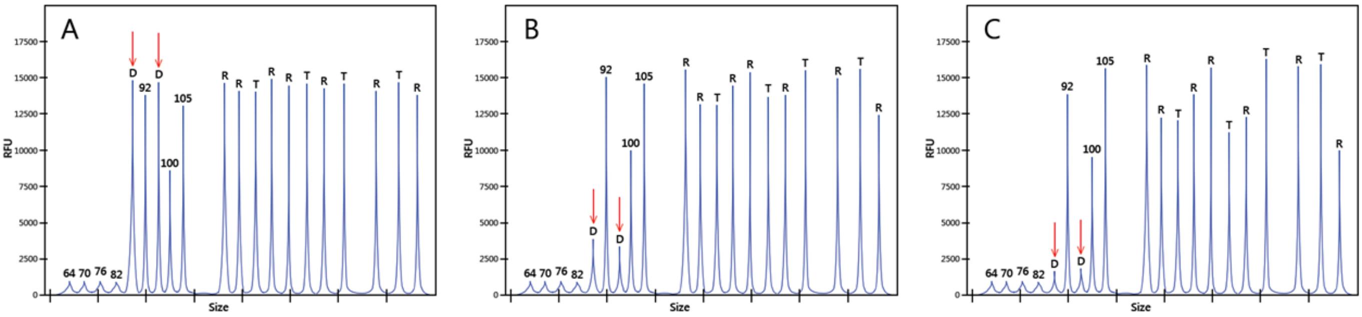

Figure 4. Effect of poor denaturation on D-fragments. D-fragments are low when sample DNA denaturation is incomplete (here induced by adding salt to the sample). MLPA results on DNA sample containing in **A.** TE, **B.** TE + 40 mM NaCl, **C.** TE + 100 mM NaCl. Samples **B.** and **C.** show insufficient denaturation.

8.2. NO-DNA CONTROL

In a typical no-DNA control, only the four Q-fragments are visible. In some probemixes, a few peaks longer than 100 nt may be visible in no-DNA controls. These non-specific peaks will not influence MLPA results when sufficient sample DNA is used, as they are outcompeted by the exponential amplification of MLPA probes MLPA probes. Notify MRC Holland in case a non-specific peak in the no-DNA control is reproducibly higher than 50% of the median height of the Q-fragments.

8.3. EVAPORATION

Evaporation can occur during (A) pipetting the ligation reaction at 54°C or (B) overnight hybridisation, and causes increased salt concentration. This can result in strong sample DNA secondary structure formation and may inhibit certain probes from binding to their target sequences. In general, plates are more prone to evaporation than strips. In case you suspect evaporation, incubate 8 µl H_2O overnight at 60°C; in the morning, >5 µl H_2O should remain. For suggestions on how to eliminate evaporation, see section 8.4 step 5. When using mineral oil, add just enough to cover the surface. There is no need to remove the oil. After probemix and polymerase master mix addition, centrifuge tubes briefly. After ligase master mix addition, pipet up and down below the oil layer.

8.4. QUALITY CONTROL FLOWCHART

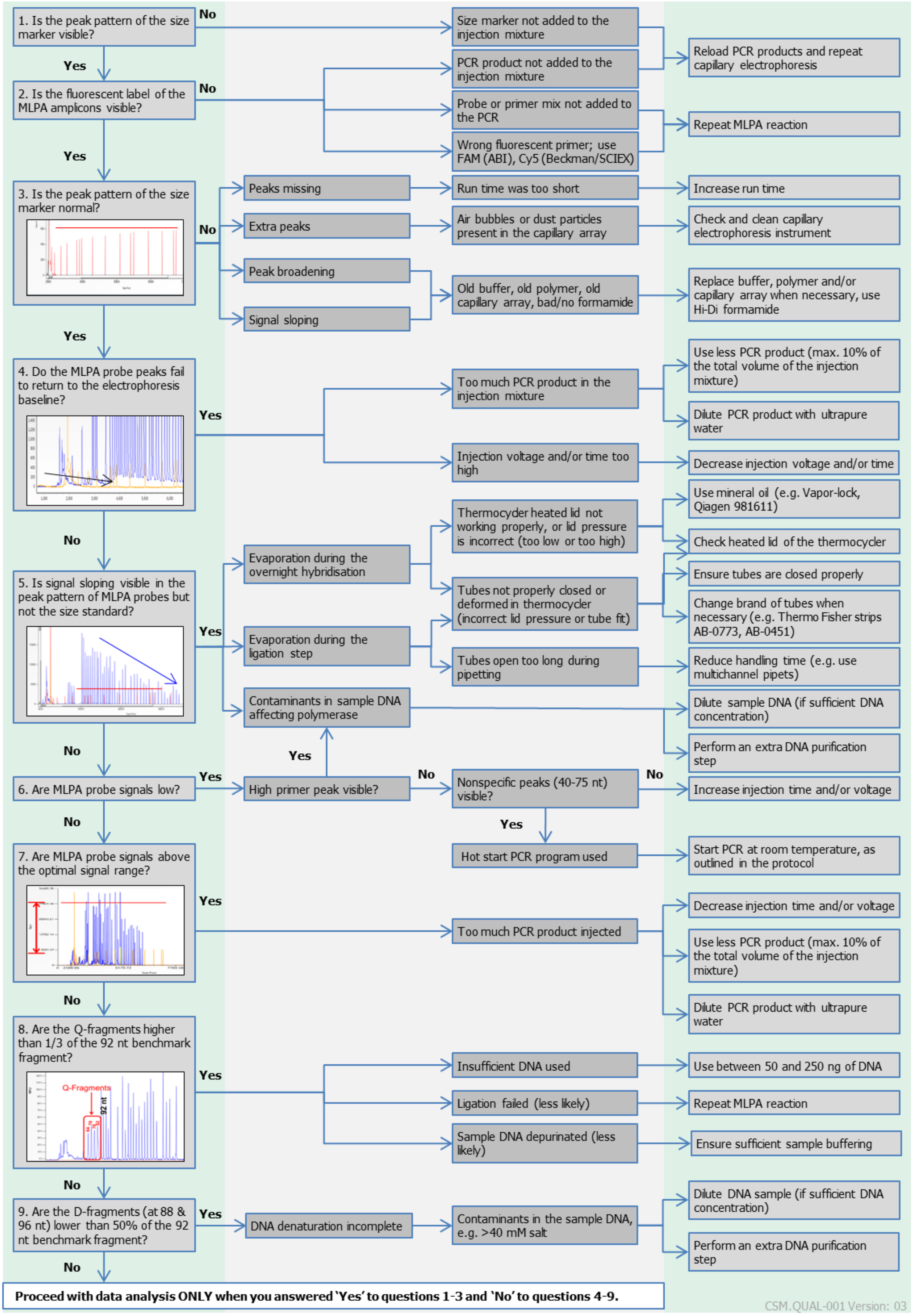

9. DATA ANALYSIS

Coffalyser.Net software, in combination with the appropriate lot-specific Coffalyser sheet, should be used for MLPA data analysis. For both, the latest version should be used. The Coffalyser.Net Reference Manual provides step-by-step instructions on MLPA data analysis. Both software and manual are freely downloadable on www.mrcholland.com.

The absolute fluorescence measured by capillary electrophoresis for each probe is affected by many variables and cannot be used directly. The fluorescence of each probe must first be normalised within an MLPA reaction. This normalisation uses reference probes that are expected to have a normal copy number in all samples. Relative probe signals from each sample are compared with those obtained from the reference samples. Reference samples are expected to have a normal copy number for all reference and target probes. This comparison then allows for the determination of the relative copy number of the target sequences in a sample.

Coffalyser.Net selects the best analysis method for each MLPA probemix and offers extensive quality control[7]. For more information on how data analysis is performed, see the Coffalyser.Net Reference Manual. **For IVD-registered MLPA applications, Coffalyser.Net must be used! Using other software may lead to inconclusive or false results!**

10. INTERPRETATION AND CONFIRMATION

- Abnormalities detected by MLPA should be confirmed by an MLPA confirmation probemix or an independent technique whenever possible. Copy number changes detected by a single probe always require confirmation. Sequencing of probe target sequences may show that a lowered probe signal is caused by a mutation/polymorphism. The finding of two heterozygous sequences typically indicates that the sample DNA contains two different alleles. Note that finding a single rare allele by sequencing does not imply that one allele is deleted, as two copies of the rare allele may be present. A homozygous SNP can lead to a partial signal reduction that resembles a heterozygous deletion.
- Not all deletions and duplications detected by MLPA are pathogenic. Germline copy number variations reported in healthy individuals can be found at http://dgv.tcag.ca/dgv/app/home. MRC Holland cannot provide information whether a deletion or duplication of a specific exon will result in disease.
- Certain copy number aberrations can be due to somatic alterations, including large deletions and duplications of entire chromosomes.
- In case of an apparent homozygous deletion, the electropherogram should be visually inspected to identify whether the signal is truly absent. Missing probe signals can be due to binning problems or low signals.
- Copy number changes detected by reference probes or flanking probes are unlikely to be related to the condition tested for. The identity of reference probes is available on request.

11. PRECAUTIONS AND WARNINGS

- For professional use only. Assay performance is dependent on operator proficiency and adherence to procedural directions. The assay should be performed by professionals trained in molecular techniques. The person responsible for result interpretation should be aware of the latest scientific knowledge of the application in question and of any limitations of the MLPA procedure that could lead to incorrect results.
- Internal validation of each MLPA application is essential, in particular when using MLPA for the first time, or when changing the sample handling procedure, DNA extraction method or instruments used; include at least 16 normal DNA samples. Validation should show a standard deviation ≤0.10 for every probe (unless the relevant probemix-specific product description states otherwise). Samples used for validation should be representative of samples used in daily practice.

12. LIMITATIONS OF THE PROCEDURE

- For most MLPA applications, the major cause of genetic defects are small (point) mutations, most of which will not be detected by MLPA probemixes.
- MLPA cannot detect any deletions or duplications that lie outside the target sequence of the probes and will not detect copy number neutral inversions or translocations.
- Sequence changes (e.g. SNPs, point mutations, small indels) in or near the target sequence detected by a probe can cause false positive results[8].

[7] Coffalyser.Net starts with raw data analysis (baseline correction, peak identification) and provides extensive quality control (e.g. DNA quantity used, complete DNA denaturation, slope correction).
[8] When designing probes, known SNPs are avoided when possible. However, new SNPs are continuously being discovered. Please notify us when a polymorphism or a frequent pathogenic mutation influences a probe signal.

- Contamination of DNA samples with cDNA or PCR amplicons of individual exons can lead to an increased probe signal[9]. Analysis of a second independently collected and isolated DNA sample can exclude these contamination artefacts.
- In case of poor sample DNA denaturation, apparent deletions, even of several probes recognising adjacent genomic targets, can be a false positive result! Extremely GC-rich chromosomal regions are not denatured at 98°C when more than 40 mM NaCl or KCl is present.
- MLPA tests provide the average copy number of the target sequences in the cells from which the DNA sample was extracted. In case several probes targeting adjacent sequences have an unusual value, but do not reach the usual threshold values for a deletion/duplication, mosaicism is a possible cause.
- Minor differences in experimental execution may affect the MLPA peak pattern. Only include samples in an analysis that were a) included in the same MLPA experiment and b) tested with the same probemix lot.
- Subtle changes, such as those observed in mosaic cases, may only be distinguished when probes are arranged according to chromosomal location.
- In certain cases, analysis of parental samples might be necessary for correct result interpretation.
- When running MLPA products, the capillary electrophoresis protocol may need optimization. False results can be obtained if one or more peaks are off-scale. For example, a duplication of one or more exons can be obscured when peaks are off-scale, resulting in a false negative result. The risk on off-scale peaks is higher when probemixes are used that contain a relatively low number of probes. Coffalyser.Net software warns for off-scale peaks while other software does not. If one or more peaks are off-scale, rerun the PCR products using either: lower injection voltage / injection time settings, or a reduced amount of sample by diluting PCR products.

MLPA General Protocol – Document History
Version-007 (01 March 2019)
- Protocol has been restructured and some sections have been rewritten. There are no changes to the method by which MLPA is performed.
- DTT has replaced Beta-Mercaptoethanol in the SALSA MLPA Buffer and SALSA Ligase-65.
- New limitation of the procedure added regarding off-scale peaks.
Version-006 (23 March 2018)
- New Figure 2 added.
- Initial settings and ABI-310 removed from electrophoresis specifications table.
- ABI-SeqStudio added to electrophoresis specifications table.
- Table with signal ranges for capillary electrophoresis instruments added.
- Quality Control Flowchart added.
- Critical points for obtaining good results added.
- Information in protocol reorganized and rewritten.

[9] Varga RE et al. (2012). MLPA-based evidence for sequence gain: pitfalls in confirmation and necessity for exclusion of false positives. *Anal Biochem.* 421:799-801.
